# Applications, image analysis, and interpretation of computer vision in medical imaging

**DOI:** 10.3389/fradi.2025.1733003

**Published:** 2026-01-09

**Authors:** Yasunari Matsuzaka, Masayuki Iyoda

**Affiliations:** 1 Department of Microbiology and Immunology, Showa Medical University Graduate School of Medicine, Shinagawa-ku, Tokyo, Japan; 2 Division of Nephrology, Department of Medicine, Showa Medical University Graduate School of Medicine, Shinagawa-ku, Tokyo, Japan

**Keywords:** application programming interfaces, computer vision, convolutional neural networks, deep learning, machine learning

## Abstract

This review summarizes the current advances, applications, and research prospects of computer vision in advancing medical imaging. Computer vision in healthcare has revolutionized medical practice by increasing diagnostic accuracy, improving patient care, and increasing operational efficiency. Likewise, deep learning algorithms have advanced medical image analysis, significantly improved healthcare outcomes and transforming diagnostic processes. Specifically, convolutional neural networks are crucial for modern medical image segmentation, enabling the accurate, efficient analysis of various imaging modalities and helping enhance computer-aided diagnosis and treatment planning. Computer vision algorithms have demonstrated remarkable capabilities in detecting various diseases. Artificial intelligence (AI) systems can identify lung nodules in chest computed tomography scans at a sensitivity comparable to that of experienced radiologists. Computer vision can analyze brain scans to detect problems such as aneurysms and tumors or areas affected by diseases such as Alzheimer's. In summary, computer vision in medical imaging is significantly improving diagnostic accuracy, efficiency, and patient outcomes across a range of medical specialties.

## 1 Introduction

Computer vision engineering is crucial to the advancement of medical imaging technologies and applications (1). This interdisciplinary field combines computer science, mathematics, and healthcare expertise to develop innovative solutions for analyzing and interpreting medical images (2). Computer vision in medical imaging encompasses several important tasks. In image classification, deep learning (DL) and convolutional neural networks (CNNs) have significantly improved medical image classification, such as identifying abnormalities in chest x-rays (CXRs) and detecting cancerous lesions (3). Image segmentation, or the division of medical images into distinct regions or objects of interest, is particularly useful in robotic surgery training and instrument segmentation during procedures. For object detection and recognition, computer vision algorithms can locate and identify specific structures, anomalies, or instruments within medical images, aiding in diagnosis and surgical planning (4). When selecting computer vision algorithms for a project, it is needed to carefully evaluate several key criteria to ensure the most appropriate solution. A comprehensive breakdown of the main selection criteria included core selection factors, such as task requirements, accuracy vs. speed trade-offs, and computational resources (5). Algorithm choice depends on three factors: task type, processing speed needs, and hardware constraints. Different algorithms excel at different tasks like classification, detection, segmentation, or feature extraction (6). Also, YOLO and ORB (Oriented FAST and Rotated BRIEF) deliver real-time performance for speed-critical applications like autonomous driving, while U-Net and Mask R-CNN provide pixel-level precision for medical imaging and segmentation tasks (7). Further, traditional algorithms (SIFT, SURF, HOG) work without training data on resource-constrained devices (8). Deep learning models like CNNs require substantial computational power, while lightweight algorithms like ORB are designed for embedded systems and mobile devices. Furthermore, as technical considerations, there are training data requirements, robustness and invariance, and feature complexity. Traditional algorithms like SIFT and edge detection do not require training data, making them suitable when labeled datasets are unavailable (9). Deep learning approaches need large amounts of annotated data but can achieve higher accuracy for complex tasks (10). SIFT offers scale and rotation invariance, making it reliable for matching tasks across different viewing conditions. Simple edge detection works well for boundary identification, while complex object detection in cluttered scenes may require sophisticated deep learning architectures like Faster R-CNN or YOLO (11). Moreover, as practical deployment factors, there are real-time requirements, interpretability, model size and memory, and development resources (12). If application needs immediate processing (autonomous vehicles, live video analysis), prioritize algorithms optimized for speed even if they sacrifice some accuracy. Some applications require understanding why the algorithm made certain decisions. Traditional methods offer more interpretable results, while deep learning models can be “black boxes” that are harder to explain. For deployment on edge devices or mobile platforms, consider the model's memory footprint and whether it can run efficiently without cloud connectivity (13). Evaluate the availability of pre-trained models, frameworks, and community support. Modern deep learning frameworks offer transfer learning capabilities that can significantly reduce development time (14). The optimal algorithm choice involves balancing these criteria based on specific use case, constraints, and priorities. Many modern applications use hybrid approaches that combine traditional and deep learning methods to leverage the strengths of both (15).

Recent developments in computer vision have greatly enhanced the capabilities of medical imaging. The use of DL techniques, particularly CNNs, has revolutionized medical image analysis, improving the processing accuracy and efficiency of complex visual data (16). Advanced algorithms have upgraded the interpretation of 3D medical imaging data, enhancing depth perception and spatial understanding in applications such as computed tomography (CT) and magnetic resonance imaging (MRI) scans (17, 18). Optimized algorithms and hardware acceleration are used to analyze medical imaging data in real time, which is crucial for applications such as live surgical guidance (19).

Despite this significant progress, several challenges remain in this area. Understanding these challenges is essential for developing robust, trustworthy, and clinically valuable AI systems. Medical image labeling is expensive, time-consuming, and requires expert participation from physicians, radiologists, and specialists. Unlike natural image analysis with large-scale labeled datasets such as ImageNet, medical image analysis faces a major challenge of lacking labeled data to construct reliable and robust models (20). Image quality, resolution, artifacts, and variability across imaging devices and protocols pose challenges for standardized workflows (21). Domain shift is widespread among different medical image datasets due to different scanners, scanning parameters, and subject cohorts (22). Existing visual backbones lack appropriate priority for reliable generalization in medical settings, with models showing over 63% average performance drop when introducing confounds (23). Five core elements define interpretability: localization, visual recognizability, physical attribution, model transparency, and actionability (24). Deep learning models are criticized for their “black-box” nature which undermines clinicians' trust in high-stakes healthcare domains (25). The direct impact of medical image analysis on patient care prioritizes accuracy and reliability, with severe consequences of adversarial attacks introducing profound ethical considerations (26). VGG16 achieved 96% accuracy on clean MRI data but dropped to 32% under FGSM attacks and 13% under PGD attacks (27). Foundation models consistently underdiagnose marginalized groups, with even higher rates in intersectional subgroups such as Black female patients.

Public datasets are available, but more comprehensive and diverse medical imaging datasets are needed to train robust models (20). Moreover, despite the potential of computer vision, the use of such applications in frontline healthcare settings is limited, indicating a research–implementation gap (28). Computer scientists, medical professionals, and healthcare institutions should closely collaborate to develop clinically relevant and applicable solutions and advance this field. Thus, computer vision in medical imaging is rapidly evolving and has immense potential to improve healthcare outcomes. As technologies and interdisciplinary collaborations strengthen, more innovative applications will improve diagnosis, treatment planning, and patient care across medical specialties. This review summarizes the current advances, applications, and prospects of computer vision in advancing medical imaging technologies.

## 2 Transformation of medical image analysis via DL

DL is revolutionizing medical image analysis by providing powerful tools that improve diagnosis, treatment planning, and patient care across multiple medical specialties (29). This transformation is evident in several key areas. In automated analysis and detection, DL algorithms, particularly CNNs, can remarkably identify and categorize anomalies in medical images automatically (17, 30). These algorithms can analyze various types of medical images, including x-rays, MRI scans, CT scans, and ultrasound images, providing healthcare professionals with fast, accurate insights. In addition, to improve accuracy and efficiency, DL models leverage large amounts of annotated data to learn complex patterns and relationships within medical images, facilitating accurate detection, localization, and diagnosis of diseases and abnormalities (17, 31). This capability enables faster, more accurate interpretation of medical images, thereby improving patient outcomes and healthcare workflow efficiency.

**Figure 1:**
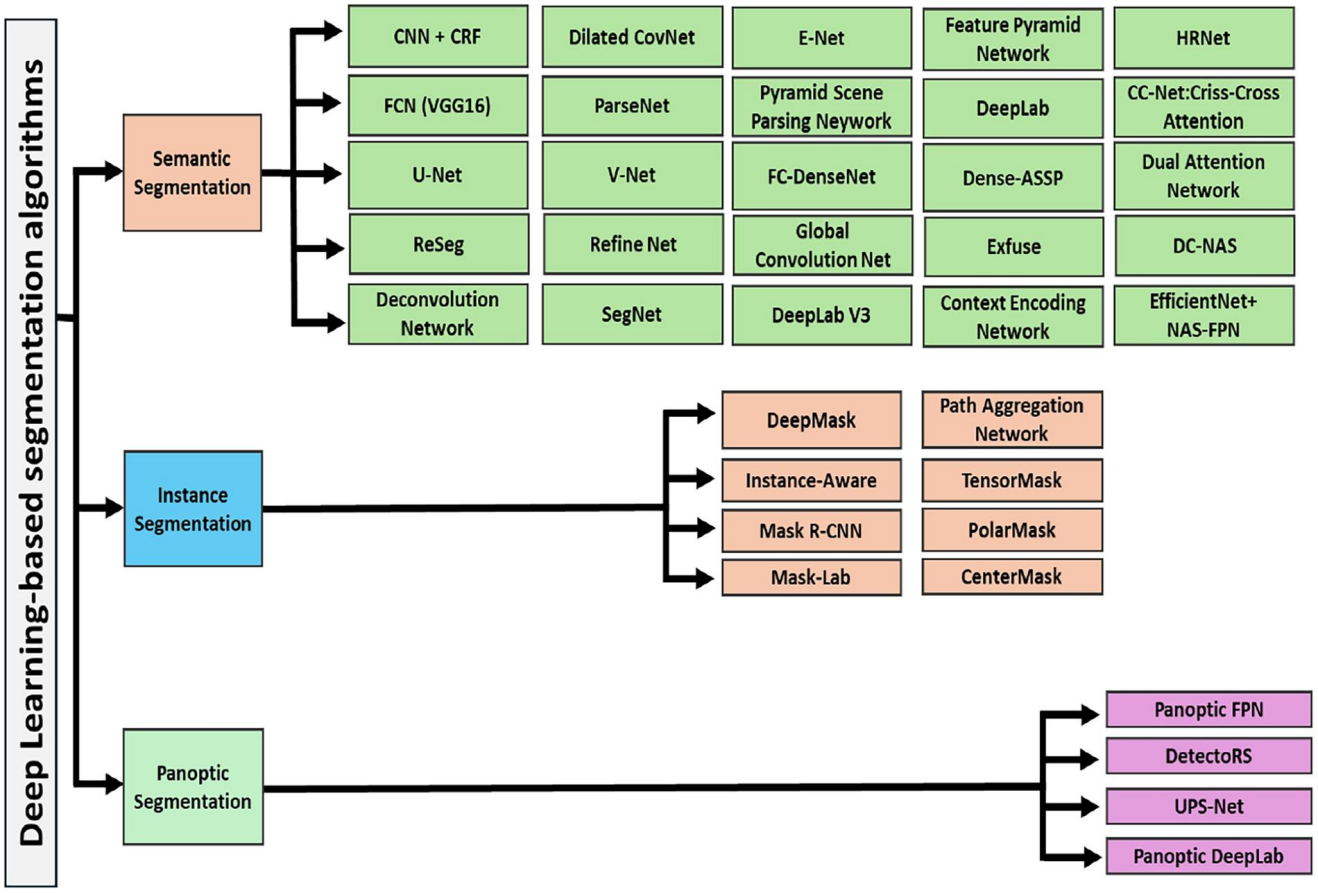
Deep learning-based segmentation algorithm. Three algorithms of the segmentation, semantic instance, and panoptic are included.

DL techniques have numerous applications in medical image analysis, such as in medical image segmentation, which is crucial for tasks such as tumor delineation; locating and identifying specific structures, anomalies, or instruments in medical images (object detection); accurately categorizing various medical conditions based on image analysis (disease classification), and improving image quality or reconstructing images from limited data (image reconstruction) (Figure 1) (32). DL algorithms are valuable tools for healthcare professionals, assisting in early disease detection, decision support for radiologists, assessment of disease progression and treatment response, and personalized treatment planning (17, 33).

The use of DL for medical image analysis is evolving, with ongoing research focusing on improving model interpretability and explainability, developing more robust and generalizable algorithms, integrating multimodal data for comprehensive analyses, and addressing privacy and security challenges (17, 34). As these advances continue, DL will be increasingly important in transforming medical imaging and healthcare delivery, ultimately leading to more personalized, accurate, and efficient patient care (29).

## 3 Main challenges in deploying computer vision in healthcare

### 3.1 Data-related challenges

Obtaining high-quality, diverse representative medical imaging datasets is difficult due to ethical, legal, and logistical issues (35). Rare diseases are often underrepresented, hindering the training of robust models. In terms of privacy and security, healthcare data are highly sensitive, exposed to high risks of data breaches and misuse (36). Strong data protection and compliance with regulations, such as the Health Insurance Portability and Accountability Act (HIPAA) and the General Data Protection Regulation (GDPR), are essential to protect personal data and maintain trust in the digital age (37). These regulations provide comprehensive frameworks for handling sensitive data, with HIPAA focusing on healthcare information in the United States and GDPR focusing on personal data protection in the European Union (EU). Both emphasize several key data protection principles: Data must be processed lawfully, fairly, and transparently (lawfulness, fairness, and transparency): data should only be collected for specific, legitimate purposes (data minimization); personal data should be kept accurate and up to date (accuracy); data should only be kept for as long as necessary (data retention); and appropriate data security measures must be taken (integrity and confidentiality).

Organizations should comply with relevant regulations by implementing robust access controls and authentication mechanisms, using encryption for sensitive data storage and transmission, and conducting regular risk assessments and security audits. They should also provide comprehensive data protection training to their staff and establish clear data handling and breach notification policies. By adhering to these principles and strengthening their data protection measures, organizations can ensure compliance with HIPAA and GDPR, protect individuals' privacy rights, and mitigate the risks associated with data breaches and unauthorized data access.

As for important regulatory philosophy differences, philosophy in United States (FDA) is risk-based pragmatism with innovation support, such as flexible pathways based on device risk and novelty, substantial equivalence concept (510(k)) enables iterative innovation, post-market surveillance increasingly data-driven (Real-World Evidence), PCCP framework represents adaptive regulation acknowledging AI's evolving nature, and “Light touch” for lower-risk devices; rigorous review for breakthrough technologies (Table 1) (38). In European Union, it is precautionary principle with fundamental rights protection, including comprehensive, cross-sectoral approach (MDR + AI Act + GDPR), high-risk AI systems subject to strict global requirements, human rights and ethical considerations central to framework, transparency and explainability legally mandated, and consumer protection paramount, even if slowing market entry. In Japan, philosophy is quality-first approach with measured innovation adoption thorough validation preferred over rapid deployment, cultural emphasis on safety and meticulous review, strong alignment with international standards (ISO, IMDRF), and preference for evidence from Japanese populations reflects local validation focus. Recent acceleration efforts (DASH for SaMD 2) show commitment to competitiveness.

**Table 1:** Medical image AI deployment: regulatory comparison (US vs. EU vs. Japan).

Regulatory aspect	United States (FDA)	European Union (MDR/IVDR + AI Act)	Japan (PMDA)
Primary regulatory bodies	Food and Drug Administration (FDA), Center for Devices and Radiological Health (CDRH)	European Commission, National Competent Authorities, Notified Bodies	Ministry of Health, Labour and Welfare (MHLW), Pharmaceuticals and Medical Devices Agency (PMDA)
Key legislation	Federal Food, Drug, and Cosmetic Act (FD&C Act); 21st Century Cures Act (2016)	Medical Devices Regulation (EU 2017/745), AI Act (EU 2024/1689), GDPR	Pharmaceuticals and Medical Devices Act (PMD Act, 2014)
Classification system	Risk-based: Class I (low), II (moderate), III (high risk)	Risk-based: Class I, IIa, IIb, III (Medical Devices); Minimal, Limited, High-risk, Unacceptable (AI Act)	Risk-based: Class I (General), II (Controlled), III (Highly Controlled), IV (Highly Controlled)
Approval pathways	510(k) (predicate device comparison), *de novo* (novel low-to-moderate risk), PMA (Premarket Approval for high-risk), Breakthrough Devices Program	CE Marking via: Self-certification (Class I), Notified Body assessment (IIa–III); Combined conformity assessment under MDR/IVDR and AI Act for high-risk AI	Todokede (notification for Class I), Ninsho (certification for Class II/III), Shonin (approval for Class III/IV)
AI-specific framework	Predetermined Change Control Plan (PCCP) finalized December 2024; allows pre-approved algorithm modifications without new submissions	AI Act (effective August 2024, full implementation by August 2027); high-risk AI systems require strict compliance including human oversight, transparency, data governance	Post-Approval Change Management Protocol (PACMP, March 2023); DASH for SaMD 2 strategy; Two-stage approval system for SaMD
Typical approval timeline	510(k): 3–6 months *de novo*: 6–12 months PMA: 12–18+ months Most AI devices use 510(k) pathway	Class IIa: 6–12 months Class IIb/III: 12–24+ months Current bottleneck: Notified Body capacity constraints	Class I: 1–2 months Class II/III: 6–12 months Class IV: 12–18 months SaMD Priority Review: 6 months (target from 2024)
Approved AI devices	1,250+ AI-enabled devices (as of July 2025); ∼712 in radiology; exponential growth from 6 (2015) to 223 (2023)	Thousands approved under MDR; exact numbers vary by member state; most radiology AI devices Class IIa or higher	Limited compared to US/EU; only 3 therapeutic apps approved by Sept 2023 vs. 50+ in US/EU; 15% increase in AI imaging device approvals 2018–2023
Post-market surveillance	Medical Device Reporting (MDR); Real-World Evidence (RWE) emphasis; Performance monitoring required under PCCP	Stringent post-market surveillance under MDR; Vigilance reporting; AI Act requires continuous monitoring of high-risk systems	Good Vigilance Practice (GVP) Ordinance; Safety management measures; Enhanced monitoring for SaMD updates
Algorithm update management	PCCP Framework (2024): - Pre-approved modifications without new submission - Must follow exact protocol - QMS documentation required - Deviations require new submission	AI Act Requirements: - Predefined change protocols - Continuous oversight required - Must maintain conformity throughout TPLC - Combined MDR/AI Act assessment	PACMP (2023): - Predefined parameters for updates - Risk-mitigated modifications - Post-approval within safety boundaries - 30-day review for IDATEN system changes
Data requirements	Clinical validation required; increasing acceptance of RWE; bridging studies may be accepted for global data	High-quality datasets mandated under AI Act; GDPR compliance required; data from EU populations often preferred	Japanese population data often required; bridging studies from global trials accepted; stricter requirements for novel devices
Transparency & explainability	Transparency Guidance (June 2024): - Human-centered design principles - User interface clarity - Model description in submissions - Not mandated but strongly recommended	AI Act Mandates: - High transparency for high-risk systems - Explainability requirements - Fundamental rights considerations - Documentation of AI decision-making process	Transparency requirements aligned with international standards (JIS T 62366-1:2022); Human factors engineering required as of April 2024
Data privacy framework	HIPAA (sector-specific): - Applies to covered entities only - Permits data sharing for treatment/payment - Separate state privacy laws - No comprehensive federal AI privacy law	GDPR (cross-sectoral): - Comprehensive data protection - Applies to all health data - Strict consent requirements - Right to explanation - Data minimization principles	Act on Protection of Personal Information (APPI): - Similar to GDPR but less stringent - Special provisions for sensitive medical data - Focus on appropriate data handling
Liability framework	Mixed liability model: - Product liability (Restatement Third of Torts) - Medical malpractice for physicians - Manufacturer responsibility unclear for adaptive AI - Case-by-case determination	Strict liability model: - Product Liability Directive (85/374/EEC) under revision - New AI Liability Directive (2024/2853) - No-fault product liability - Burden of proof on manufacturer - Penalties under AI Act	Mixed liability model: - Product Liability Law - Medical malpractice framework - Manufacturer accountability for defects - Healthcare provider responsibility for clinical use
Human oversight requirements	Recommended but not mandated; clinical decision support software must allow independent physician review	AI Act Mandates: - Human oversight required for high-risk AI - Cannot fully replace human judgment - Override capability necessary - Recognized as risk mitigation factor	Encouraged through human factors engineering requirements; physician final decision-making authority maintained
Documentation language	English	Native languages of member states + English increasingly accepted	Japanese required (all documentation); English submissions under pilot for certain applications (as of Sept 2024)
International harmonization	Participates in IMDRF (International Medical Device Regulators Forum); collaborative guidance with UK MHRA and Health Canada	Leader in international AI governance; IMDRF participation; AI Act influences global standards	Active IMDRF participant; aligns with ICH, ICMRA, MDSAP; ISO 13485 compliance
Innovation support mechanisms	- Breakthrough Devices Program (accelerated review) - Pre-submission meetings - Q-Submission program - Real-World Evidence pilots	- Innovation support via EU funds - Regulatory sandboxes (member state dependent) - SME support initiatives - Notified Body guidance	- DASH for SaMD 2 program - Priority review for SaMD - Two-stage approval system - Pre-submission consultations - Expanded review team for SaMD
Key challenges	- 510(k) predicate pathway complexity - Unclear guidance for truly novel AI - Time-intensive for complex devices - Post-market surveillance requirements	- Notified Body capacity constraints - Dual compliance (MDR + AI Act) - Regulatory fragmentation across member states - Lengthy certification timelines - High compliance costs	- Language barrier (Japanese documentation) - Japanese population data requirements - Slower adoption than US/EU - Limited number of approved SaMD - Rigorous QMS requirements
Deployment timeline impact	Fast-to-Market: - 510(k) enables rapid market entry - Established predicate pathways - Largest approved device database - Iterative updates via PCCP	Moderate Pace: - CE marking timeframe variable - Notified Body availability critical - Dual assessment (MDR + AI Act) adds complexity - Single market access advantage	Deliberate Pace: - Focus on quality over speed - Comprehensive review process - SaMD priority review improving timelines - Cultural emphasis on thorough validation
Clinical trial requirements	- Pivotal clinical trials for PMA pathway - May accept foreign clinical data - Good Clinical Practice (GCP) compliance - IDE required for investigational devices	- Clinical evaluation required under MDR - Clinical Trials Regulation (CTR) - GCP compliance mandatory - May require EU-specific data	- GCP compliance required - Often requires Japanese population data - Bridging studies common - Clinical data from outside Japan may be accepted with justification
Bias & fairness requirements	Emphasis on bias mitigation in PCCP guidance; recommendations for diverse datasets; no explicit mandates	AI Act requires: - High-quality, representative datasets - Bias monitoring and mitigation - Fundamental rights assessment - Population diversity considerations	Aligned with international standards; increasing focus on data quality and representation
Cybersecurity requirements	FDA cybersecurity guidance; premarket and postmarket requirements; Software Bill of Materials (SBOM)	Cyber Resilience Act; NIS2 Directive; cybersecurity mandatory for high-risk AI systems	Aligned with international cybersecurity standards; QMS includes cybersecurity provisions
Cost implications	Moderate: - 510(k): $10,000-$50,000 - *de novo*: $50,000-$150,000 - PMA: $200,000-$1M+- User fees + compliance costs	High: - Notified Body fees: €50,000-€300,000+ - Dual compliance (MDR + AI Act) - Multiple market authorizations if multi-state - Legal/consulting fees substantial	Moderate-High: - Translation costs significant - Japanese representative/MAH required - Consultant fees for navigation - QMS certification costs
Market access strategy	Single approval for entire US market (330M+ population); largest single medical device market	Single CE mark grants access to 27 EU member states (450M+ population); some national requirements remain	Third-largest medical device market ($30B by 2025); gateway to broader Asia-Pacific region

### 3.2 Technical challenges

**Figure 2:**
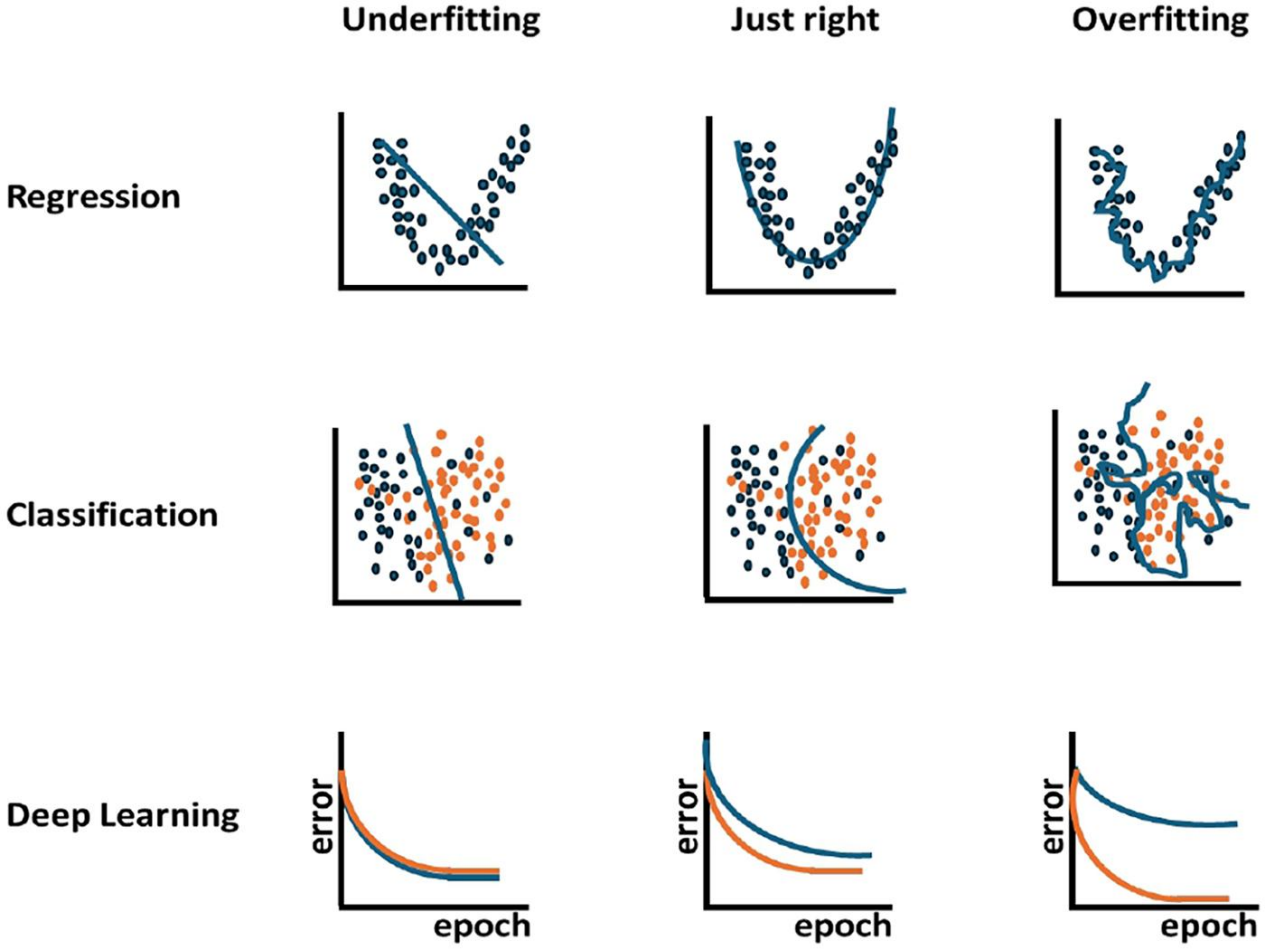
Underfitting and overfitting in regression, classification, and deep learning. (middle) Blue and orange dots represent classes in binary classification. (bottom) In deep learning, orange lines are training datasets, and blue lines are validation datasets.

Artificial intelligence (AI) models in healthcare often struggle to generalize across different patient populations and healthcare settings (39) due to several factors, including variations in equipment, procedures, and patient demographics. AI models often perform inconsistently across demographic groups (40). For example, models trained primarily on middle-aged adults may inaccurately diagnose conditions in pediatric or geriatric populations due to differences in imaging characteristics and health profiles that are underrepresented in their training datasets (41). In addition, different hospitals and clinics may use varying protocols, equipment, and data collection methods (42). A recent study highlighted the effect of sample size and patient characteristics, such as age, comorbidities, and insurance type, on the performance of a clinical language model (ClinicLLM) trained on data from multiple hospitals. The results showed that models often generalized poorly in hospitals with smaller samples or for patients with certain insurance types (43). The insufficient representation in training datasets also significantly limits the generalizability of AI models (data representation issues) (44). Privacy constraints often prevent access to comprehensive datasets that include diverse demographics, leading to biases, which can compromise model performance in different populations (45). In addition, when a model learns too much about the training data, including noise and anomalies, its ability to generalize new data is impaired (overfitting and model uncertainty) (Figure 2) (46). This issue is particularly relevant in clinical settings, where the variability of patient conditions can differ considerably from that of the training environment.

Several strategies can be used to improve the generalization capabilities of AI models in healthcare (47). Models can be tailored to specific hospital settings (local fine-tuning) to improve their adaptability to the unique characteristics of the patient population in each facility, thus improving model performance. In addition, the ranges of patient demographics and health conditions in training datasets can be broadened to mitigate bias and improve overall model performance in diverse settings (diverse data inclusion) (48). In addition, AI systems should be assessed constantly for biases and disparities (continuous monitoring and adaptation) (49). User feedback should be used to refine AI technologies continuously and ensure they remain effective in evolving cultural contexts and patient needs (50). In summary, addressing the challenges of model performance and generalization in healthcare AI requires a multifaceted approach that considers the complexities of different patient populations and healthcare environments. The effectiveness of AI applications in healthcare can be considerably improved by focusing on local adaptations, inclusive data practices, and continuous evaluation.

Integrating new computer vision systems into healthcare IT infrastructure is challenging due to the complexity and siloing of existing systems (51). Healthcare organizations often operate with fragmented data across different departments and systems (52). This isolation hinders information sharing, leading to inefficiencies in patient care and decision-making. Approximately 80% of healthcare data are unstructured and reside in disparate systems, worsening the problem of data silos (53). Furthermore, many healthcare providers rely on outdated technologies that were not designed for interoperability. These legacy systems increase the difficulty of integrating new technologies, such as computer vision systems, because they often cannot communicate effectively with modern applications. In addition, the lack of standardized protocols and the wide variety of data formats prevent seamless communication between different healthcare systems (54). Certain regulations also mandate strict privacy measures during information transfer (interoperability issues). Moreover, the integration of new technologies requires a robust IT infrastructure capable of supporting complex data exchange (technical barriers) (55). Without modern, flexible systems that can accommodate such integration, healthcare organizations face significant hurdles in achieving interoperability (56).

These integration challenges can be overcome using certain strategies. Implementing standards such as Fast Healthcare Interoperability Resources can enhance communication between different systems for manageable integration (adopting standards) (57). In addition, application programming interfaces (APIs) can enable different software applications to communicate effectively for the smooth integration of new technologies with existing systems (use of APIs). Furthermore, investing in comprehensive data platforms that centralize data management can help eliminate silos and improve interoperability between different healthcare applications (integrated data platforms) (58). Thus, integrating new computer vision systems into complex, siloed healthcare IT infrastructure requires a solution to challenges related to legacy systems, interoperability, and regulatory compliance. Strategies such as the use of standardized protocols and APIs can help facilitate seamless integration.

Computer vision applications require combining software algorithms and specialized hardware, presenting challenges for optimal implementation (59). Moreover, implementing computer vision systems can be expensive due to their need for powerful hardware and complex software setups. This often entails hiring skilled personnel for development and maintenance, incurring added costs. The adaptability of computer vision systems can be both a strength and a challenge (60). Although they can learn from data, they also require large datasets for training and regular updates to maintain their performance in dynamic environments. Therefore, computer vision applications can be implemented successfully by ensuring the balanced integration of advanced hardware and sophisticated software algorithms and considering the challenges posed by resource requirements and system complexity.

### 3.3 Ethical and interpretability challeng**es**

DL models, often called black boxes, present critical challenges regarding explainability and interpretability (61). These challenges can hinder trust and adoption, particularly in sensitive domains, such as clinical settings, where decisions can profoundly affect patient care. The term “black box” refers to the opaque nature of DL models; their internal decision-making processes are not easily understood by humans (62). This lack of transparency increases the difficulty of diagnosing problems when such models produce unexpected or harmful results. For example, when an autonomous vehicle fails to stop for a pedestrian, the reason for this decision of the model is nearly impossible to understand due to its complex internal workings (63). This opacity can lead to misplaced trust in AI systems, as users may be unable to determine the reliability or fairness of such systems' decisions.

Explainability is the provision of clear reasons for AI models' decisions, allowing users to understand why particular results are produced (64). Interpretability refers to understanding how a model processes inputs to arrive at its outputs. Both concepts are critical in domains such as healthcare, where understanding the rationale behind a model's decisions can directly affect patient outcomes. An interpretable model allows users to understand its decision-making process, promoting accountability and transparency (responsibility) (65). When users understand how a model works and why it makes certain decisions, their trust in AI systems increases substantially (trust) (64). Several methods can be used to address the black-box problem. Tools such as heat maps can illustrate the features that most influence a model's decisions (visualization techniques). In addition, breaking down complex models into simpler components can help clarify how decisions are made (decomposition methods). Providing users with examples like their input can also explain how a model arrives at its conclusions (example-based explanations). The black-box nature of DL models is therefore a significant barrier to their adoption in critical applications, such as healthcare (66). Their explainability and interpretability should be improved to foster trust and ensure their responsible use. As AI evolves, transparency should be prioritized for its successful integration into high-stakes environments.

Automated decision-making in healthcare, particularly using AI, raises ethical concerns (67), which primarily revolve around bias, accountability, and its effect on patient autonomy. Algorithmic bias, which can occur in different stages of AI development, including data collection, model training, and development, is a crucial ethical problem (40). Biased data can lead to suboptimal clinical decisions and exacerbate healthcare disparities, particularly affecting marginalized populations (68, 69). For example, AI systems trained on nonrepresentative datasets may produce recommendations that inadequately serve patient groups, leading to inequitable treatment outcomes (68). In addition, automation bias, where healthcare providers may rely excessively on AI recommendations, may cause errors of omission or commission. This bias can result from cognitive complacency, or clinicians choosing to rely on automated systems rather than exercising their clinical judgment (70). Such reliance can be dangerous, particularly if the AI system produces incorrect or misleading results.

Accountability is critical in the context of AI-driven healthcare decisions. When an AI system makes a mistake, such as an incorrect diagnosis, questions arise about the responsible party: the algorithm developers, healthcare providers (system users), or regulatory bodies (overseers of its implementation) (71). These algorithms' lack of transparency further complicates the problem. Many AI systems operate as black boxes, so the bases for their recommendations are difficult for clinicians to understand (72). This opacity can undermine the trust between healthcare providers and patients. These accountability concerns can be addressed by establishing clear frameworks that delineate the responsibilities of stakeholders involved in AI use (73). These obligations include rigorously testing algorithms, regularly monitoring their accuracy, and ensuring that healthcare providers retain the ultimate decision-making authority.

Automated decision-making also poses challenges related to patient autonomy. Patients should be informed about how AI will influence their care decisions and be allowed to participate in discussions about their treatment options (74). AI systems' prioritization of certain medical outcomes over individual patient preferences, such as quality of life over survival rates, can undermine patient autonomy and lead to their dissatisfaction with care (75). In addition, ensuring that patients understand the implications of AI involvement in their care is essential for informed consent (76). Patients should be aware of the prospective use of their data and the potential risks associated with automated decision-making (69). Thus, although automated decision-making in healthcare has the transformative potential to improve patient outcomes and operational efficiency, it presents complex ethical challenges. Addressing biases in AI systems, clarifying their accountability structures, and safeguarding patient autonomy are necessary to ensure that these technologies are implemented ethically and effectively (77). Developers, healthcare providers, regulators, and patients should cooperate in navigating these ethical concerns to foster trust and improve the quality of care delivered using AI.

### 3.4 Validation and regulatory challenges

Clinical validation of computer vision systems in healthcare is a complex, time-consuming process needed to ensure quality, safety, and efficacy (51). It entails thorough tests in real-world clinical settings, such as interventional or clinical trials evaluating the performance of AI systems, comparisons with relevant systems and assessment of meaningful endpoints, and minimization of bias in study design and implementation (78). Validation includes the reporting of performance metrics in internal and independent external test data and the benchmarking of system performance against care standards and other AI systems (79). Evaluation should continue after the initial validation. Performance and effects on care, including safety and effectiveness, should be monitored to understand expected and unexpected outcomes. Algorithmic audits should be conducted to understand the mechanisms of adverse events or errors. Several factors contribute to the complexity and considerable time consumption of clinical validation. Ensuring the high quality, diversity, and representativeness of data for AI model training and testing is challenging (data quality). Furthermore, the seamless integration of such models into existing healthcare systems and workflows can be difficult (interoperability and integration) (80). The evolving regulatory landscape for AI-based medical devices is likewise an ongoing challenge (regulatory compliance) (56), and validating AI systems in different clinical settings and patient populations is critical but complex (real-world performance) (81). Addressing bias, fairness, and patient privacy problems adds a layer of complexity to validation (ethical considerations). Despite these challenges, rigorous clinical validation is essential to build confidence in AI-based computer vision systems and ensure their safe, effective implementation in healthcare (82).

Meeting the stringent regulatory requirements for medical devices and AI in healthcare is indeed challenging and resource intensive. The continuous evolution of regulations requires constant vigilance and adaptation (ever-evolving regulations) (83). Documentation requirements, such as device master and design history files, are difficult to comply with (complex documentation), and compliance strategies need to be harmonized to navigate different international regulatory frameworks (global market variability). Regulating AI in healthcare presents unique difficulties, including data governance and bias mitigation, ensuring AI system transparency and accountability, and implementing effective risk management and postmarket surveillance (83). Moreover, keeping up to date with regulations and maintaining compliance requires considerable time and financial investment (resource constraints). Digitization of medical devices raises additional security concerns related to patient data protection (cybersecurity concerns). Regulators also must encourage innovation while ensuring patient safety and device efficacy (balancing innovation and safety). Regulators are adapting their approaches to address these challenges. For example, the EU AI Act uses a risk-based approach to regulate AI systems, categorizing them according to potential risk level (83). In addition, some regulators are exploring the use of AI itself to improve compliance processes, such as using machine learning (ML) algorithms to analyze clinical and operational data in real time (77).

### 3.5 Practical implementation challenges

Obtaining expert annotations for medical images is difficult due to financial and time costs (84), among other factors. Its need for specialized equipment, software, and highly skilled personnel incurs high costs, especially for small medical institutions (85). Furthermore, depending on the complexity of regions of interest and local anatomical structures, annotating an image can take minutes to hours (86). Medical images can be large, with some scans generating gigabytes of data (87), making their annotation process resource intensive and difficult to manage effectively. Time constraints often prevent medical professionals from allocating sufficient time for image annotation, especially in cases requiring rapid diagnosis and treatment (88). Medical images can be complex, requiring interpretation by highly skilled experts trained in specific medical imaging techniques (89). Moreover, applying DL to medical image analysis requires unprecedented amounts of labeled training data, incurring financial and time costs (large datasets) (90). These factors constitute a bottleneck in the development of AI-based medical imaging and limit the clinical applicability of DL.

The performance of ML models often decline gradually over time—a phenomenon called model decay, or AI aging (91). A recent study by researchers from the Massachusetts Institute of Technology, Harvard, and other institutions found that 91% of ML models degrade over time (92). This degradation is due to several factors, including temporal changes in the statistical properties of input data (data drift), relationship changes between input and output variables (concept drift), and a temporal decline in performance since model training (model aging). These issues should be addressed via continuous model monitoring and updating. Continuous model monitoring involves tracking of performance metrics and model behavior under real-world conditions; detection of data drift, concept drift, and outliers; and analysis of the root causes of performance problems. By implementing these practices, organizations can proactively manage model decay and maintain the performance reliability of their models over time.

Computer vision systems benefit healthcare, but healthcare professionals require extensive training to use and interpret them effectively (51). Such technology also requires proper understanding and integration into clinical workflows to improve diagnostic accuracy and efficiency (93). Healthcare professionals need to understand the basics of computer vision algorithms, including their capabilities and limitations (94). Consequently, they will be able to interpret results appropriately and avoid overreliance on automated systems. Training should focus on interpreting the outputs of computer vision systems, especially for complex medical images, such as x-rays, MRIs, and CT scans (17). Professionals should combine algorithmic insights with their clinical expertise to optimize patient care (95). Healthcare workers need training to integrate computer vision tools into their daily routines for seamless adoption and maximum efficiency gains (96). Their training should include the use of software interfaces and incorporation of system insights into decision making. As computer vision technology rapidly evolves, ongoing training is essential to keep healthcare professionals abreast of new developments and applications (97). Consequently, they can use recent advances to improve patient outcomes. By investing in comprehensive training programs, healthcare institutions can maximize the benefits of computer vision technology while maintaining high standards of patient care and safety (96). Addressing the abovementioned challenges entails collaboration between healthcare providers, AI researchers, regulators, and other stakeholders to develop robust, ethical, clinically relevant computer vision solutions for healthcare.

## 4 Improvement in medical image segmentation using CNNs

### 4.1 CNN models

CNNs have considerably improved medical image segmentation. They can automatically learn and extract relevant features from medical images, overcoming the limitations of traditional algorithms, which rely on manually designed features (automatic feature extraction) (98). DL-based CNN models have state-of-the-art accuracy in various medical image segmentation tasks, often at levels comparable to those of expert radiologists (improved accuracy) (87). CNNs use key formulas for image segmentation. A fundamental operation in CNNs is convolution, which is expressed mathematically as

$$(f*g)(x,y)=\sum_{i\;=\;-a}^{a}\sum_{j\;=\;-b}^{b}f(i,j)\;\cdot \;g(x-i,y-j)$$ (1)

where ***f*** is the input image, ***g*** is the kernel or filter, and ***x*** and ***y*** are the output pixel coordinates.

Another important formula in CNNs for image segmentation is the activation function, commonly a rectified linear unit (ReLU), which is expressed mathematically as

ReLU(x)=max(0,x) (2)

This function allows the network to learn complex patterns by introducing nonlinearity.

Max pooling is often used for pooling operations that reduce the spatial dimensions of feature maps.

MaxPool(X)=max(xi,j) (3)

where ***X*** is a subregion of the feature map. These formulas work together in CNNs to learn features, reduce dimensionality, and segment images into meaningful regions or objects.

Advanced CNN architectures, such as U-Net, can capture both local and global image features for the accurate segmentation of complex anatomical structures (multiscale feature learning) (98, 99). U-Net is widely used for image segmentation tasks. Its key formulas are as follows:

Convolutional layers:

y=f(W∗x+b) (4)

where ***y*** is the output, ***x*** is the input, ***W*** is the conventional kernel, ***b*** is the bias, and ***f*** is the activation function (typically ReLU).

Upsampling:

$$x_{1}\;=\;UpSample(x)$$ (5)

$$x\;=\;Concat(x_{1},x_{2})$$ (6)

where ***x*_1_** is the unsampled feature map, ***x*_2_** is the skip connection from the encoder, and ***Concat*** is the concentration operation.

Finalconvolution:y=σ(W*x+b) (7)

where *σ* is typically a sigmoid (softmax) activation function for binary segmentation (multiclass segmentation). Together, these formulas form a U-shaped architecture that allows U-Net to capture both local and global features for accurate image segmentation.

Modern CNNs can use 3D image information for a comprehensive analysis of volumetric medical imaging data, such as MRIs and CT scans (3D image processing) (87, 98). Fully convolutional architectures enable end-to-end learning for pixel-wise classification, improving overall segmentation. CNNs are also versatile, applicable to different medical imaging modalities and segmentation tasks through transfer learning (adaptability) (98). Advanced CNN models can segment images with multiple objects, occlusions, or background noise for enhanced accuracy in challenging clinical scenarios (handling of complex cases). Using these capabilities, CNNs have become a cornerstone of modern medical image segmentation, enabling accurate, efficient analyses of medical image data in various clinical applications.

### 4.2 U-Net for medical image segmentation

U-Net has revolutionized biomedical image segmentation (100). It is the most widely used image segmentation architecture in medical imaging due to its flexibility, optimized modular design, and success in various applications. The U-shaped architecture of U-Net consists of a contracting encoder path and an expanding decoder path (101). High-resolution features from the contracting path are combined with unsampled features using skip links, enabling precise localization. Furthermore, U-Net uses limited training data efficiently through extensive data augmentation, and arbitrarily large images are seamlessly integrated using the overlap tile strategy (102). Other advantages of U-Net include its accurate segmentation of small targets, and performance superiority to previous methods in various biomedical image segmentation challenges (100).

Since its inception, U-Net has influenced the study of many variations and improvements. 3D U-Net extends its architecture to handle 3D volumetric data, which is crucial for analyzing biomedical image stacks (103). Attention mechanisms enhance feature selection and segmentation accuracy (104). Dense modules improve feature reuse and gradient flow. Feature enhancement techniques are used to extract meaningful features from medical images. The loss function is improved to optimize network performance for specific segmentation tasks. Generalization enhancements have been implemented to improve the model's ability to work across different medical imaging modalities. Certain variations were developed to address the handling of different imaging modalities, improve accuracy for small or complex structures, and optimize segmentation performance with limited data. U-Net and its variants have been successfully applied to various medical image segmentation tasks, including neuronal structure segmentation in electron microscopy stacks, cell tracking in light microscopy, caries detection in dental radiography, and organ and tumor segmentation, in various imaging modalities (105). The continued development and refinement of U-Net-based architectures has significantly advanced the field of medical image segmentation, providing powerful tools for automated analysis and diagnosis in healthcare (106).

### 4.3 Comparison of U-Net with other segmentation algorithms

U-Net is the most widely used and successful image segmentation architecture in medical imaging due to its flexibility, optimized modular design, and effectiveness across different modalities (104). Compared with other segmentation algorithms, U-Net offers several advantages in aspects such as performance, efficiency, versatility, adaptability, robustness, and computational efficiency. U-Net consistently outperforms existing methods in various biomedical image segmentation tasks (107). It performs high-accuracy retinal layer segmentation in optical coherence tomography (OCT) images, often matching or exceeding the performance of more complex variants (e.g., B1). U-Net trains rapidly and can work effectively with very few training images (85), which is particularly beneficial in medical imaging, where large, annotated datasets are often scarce. U-Net has also been successfully applied to a wide range of medical imaging tasks, including neural structure segmentation, cell tracking, caries detection, and organ and tumor segmentation, across different imaging modalities (108). Since its introduction, U-Net has influenced the development of numerous variants and enhancements, allowing researchers to adapt its architecture to specific medical imaging challenges (109). These variations address problems such as the handling of 3D volumetric data, incorporation of attention mechanisms, and improvement of feature extraction.

Vanilla U-Net often performs comparably to more complex variants across different datasets and pathologies, suggesting its robustness and generalizability (109). Some U-Net variants offer marginal performance improvements while increasing complexity and slowing down inference. The original U-Net architecture balances performance and computational efficiency well (110). Thus, its combination of accuracy, efficiency, and adaptability has made it a preferred choice for medical image segmentation tasks. Its success in various applications and datasets, coupled with its ability to perform well with limited training data, distinguishes it from many other segmentation algorithms in medical imaging.

U-Net has remained a dominant architecture for image segmentation since its introduction in 2015, particularly excelling in medical imaging despite the emergence of more complex models like transformers (111). Understanding why U-Net continues to offer specific advantages requires examining its architectural design principles, computational characteristics, and practical deployment considerations (112).

#### 4.3.1 The skip connection architecture: preserving spatial information

The defining feature of U-Net is its skip connections, which directly address a fundamental problem in encoder-decoder architectures (113). Skip connections mitigate loss of fine-grained details by allowing the decoder to access high-resolution feature maps from the encoder, helping reconstruct the segmentation map with greater precision and ensuring fine details are preserved (114). U-Net is an encoder-decoder segmentation network with skip connections, where an encoder extracts more general features the deeper it goes, while skip connections reintroduce detailed features into the decoder (115). This architecture enables U-Net to segment using features that are both detailed and general.

#### 4.3.2 Bridging the semantic gap

The underlying hypothesis behind improved U-Net variants is that models can more effectively capture fine-grained details when high-resolution feature maps from the encoder are gradually enriched before fusion with semantically rich feature maps from the decoder (116). Skip connections in U-Net directly fast-forward high-resolution feature maps from encoder to decoder, resulting in concatenation of semantically dissimilar feature maps (117). This semantic dissimilarity is both a limitation and an advantage. While advanced variants like UNet++ address this through nested skip connections, standard U-Net's simpler approach often proves sufficient for many medical imaging tasks where preserving fine anatomical boundaries is critical (111).

#### 4.3.3 Gradient flow and training stability

Skip connections improve gradient flow during backpropagation, as gradients can diminish when propagated through deep networks—the vanishing gradient problem. This property makes U-Net easier to train than deeper architectures, particularly important when working with limited medical imaging datasets where training stability is paramount.

#### 4.3.4 Computational efficiency: the practical advantage

U-Net's efficiency stems from its relatively modest computational requirements compared to modern transformer-based models, making it ideal for resource-constrained medical environments (118).

#### 4.3.5 Parameter and FLOPs analysis

SD-U-Net requires approximately 8× fewer FLOPs compared to U-Net, is 81 milliseconds faster in prediction speed for 256 × 256 × 1 inputs on an NVIDIA Tesla K40C GPU, and is 23× smaller (119). This demonstrates that even the baseline U-Net is significantly more efficient than many modern architectures. Lightweight U-Net variants reduce parameters to 12.4% and FLOPs to 12.8% of standard U-Net while increasing inference speed by nearly 5 times, showing that U-Net's architecture is amenable to further optimization without sacrificing performance. U-Net model capacities range from 1.4M to 137M parameters with corresponding FLOPs computed for 128 × 128 × 128 inputs, demonstrating that optimal architecture is highly task-specific with smaller models often performing competitively (120).

#### 4.3.6 The “bigger is not always better” paradigm

The “bigger is better” paradigm has significant limitations in medical imaging, as optimal U-Net architecture is highly dependent on specific task characteristics, with architectural scaling yielding distinct benefits tied to image resolution, anatomical complexity, and segmentation classes (120). Research reveals three key insights: increasing resolution stages provides limited benefits for datasets with larger voxel spacing; deeper networks offer limited advantages for anatomically complex shapes; and wider networks provide minimal advantages for tasks with limited segmentation classes. This task-aware approach enables better balance between accuracy and computational cost.

#### 4.3.7 Data efficiency: excelling with limited samples

Medical imaging faces a chronic data scarcity problem, and U-Net's architecture is particularly well-suited for learning from limited samples (121).

#### 4.3.8 Few-shot learning compatibility

Few-shot learning techniques have been successfully adapted to rapidly generalize to new tasks with only a few samples, leveraging prior knowledge, with MAML demonstrating high performance and generalization even on small datasets (122). In 10-shot settings, enhanced 3D U-Net with MAML achieved mean dice coefficients of 93.70%, 85.98%, 81.20%, and 89.58% for liver, spleen, right kidney, and left kidney segmentation respectively (122). U-Net's success in few-shot scenarios stems from its efficient parameter usage and strong inductive biases for spatial hierarchies (123). U-Net is like auto-encoder, learning latent representation and reconstructing output with the same size as input, providing strong feature extraction capability essential for medical image segmentation (124).

#### 4.3.9 No pre-training required

Unlike Vision Transformers that require massive pre-training datasets to achieve competitive performance, U-Net can be trained effectively from scratch on small medical imaging datasets (125). This makes it particularly valuable when domain-specific data is limited and transfer learning from natural images provides limited benefits.

#### 4.3.10 Robustness considerations: the skip connection trade-off

Recent research has revealed important nuances about U-Net's robustness characteristics that highlight when its advantages are most pronounced (126). Skip connections offer performance benefits, usually at the expense of robustness losses, depending on texture disparity between foreground and background and the range of texture variations present in the training set. Training on narrow texture ranges harms robustness in models with more skip connections, while the robustness gap between architectures reduces when trained on larger texture disparity ranges (127). This finding suggests that U-Net excels when: (1) training data encompasses diverse texture variations, (2) texture disparity between target and background is moderate, or (3) the task prioritizes pixel-level accuracy over robustness to texture perturbations (128). For robustness-critical applications, careful consideration of skip connection design is warranted (129).

#### 4.3.11 Architectural simplicity: maintainability and interpretability

U-Net's straightforward encoder-decoder structure with skip connections offers advantages that extend beyond raw performance metrics (130).

#### 4.3.12 Ease of implementation and modification

The architecture's simplicity makes it easy to implement, debug, and modify for specific applications. Researchers can readily adapt U-Net by changing the encoder backbone, adjusting network depth, or incorporating attention mechanisms without fundamentally altering its core structure (131). UNet++ introduces redesigned skip connections that enable flexible feature fusion in decoders, an improvement over restrictive skip connections in U-Net that require fusion of only same-scale feature maps (115). The fact that improvements can be readily incorporated into the U-Net framework demonstrates its architectural flexibility (125).

#### 4.3.13 Interpretability

Compared to transformer-based models, U-Net's convolutional operations and skip connections are more interpretable (98). Researchers can visualize feature maps at different encoder and decoder levels, understand what spatial features are being preserved through skip connections, and identify which resolution stages contribute most to segmentation accuracy (132).

#### 4.3.14 Domain-specific advantages in medical imaging

U-Net was originally designed for biomedical image segmentation, and its architecture embodies several design choices particularly suited for medical imaging tasks (120).

#### 4.3.15 Handling class imbalance

Medical images often have severe class imbalance with diseased tissue occupying small regions (133). U-Net's architecture, combined with appropriate loss functions like Dice loss, handles this imbalance effectively (134). The skip connections ensure that small target regions maintain adequate representation throughout the network.

#### 4.3.16 Boundary precision

Skip connections help preserve spatial accuracy by bringing forward detailed features from earlier layers, especially useful when models need to distinguish boundaries in segmentation tasks (135). In medical imaging, accurate delineation of tumor boundaries or organ edges is often more critical than achieving the highest overall pixel accuracy (136).

#### 4.3.17 Multi-scale feature integration

Dense and nested skip connections aim to enhance information exchange between encoder and decoder layers, allowing comprehensive exploration of multi-scale features (115). This multi-scale capability is essential for medical images where pathologies can appear at vastly different scales.

#### 4.3.18 Practical deployment: point-of-care and edge devices

The transition of medical imaging from laboratory settings to bedside environments creates unique deployment requirements where U-Net excels. LV-UNet model sizes and computation complexities are suitable for edge device and point-of-care scenarios (137). Lightweight U-Net variants can run on resource-constrained devices including mobile phones, portable ultrasound machines, and edge computing platforms without requiring cloud connectivity or high-end GPUs (138). Segmentation of a 512 × 512 image takes less than a second on a modern GPU using U-Net architecture, enabling real-time clinical applications (139). This speed, combined with low memory requirements, makes U-Net ideal for interactive segmentation tools where clinicians need immediate feedback.

#### 4.3.19 When U-Net remains the optimal choice

U-Net offers specific advantages that make it the preferred architecture as follows (106);

(1) Limited Training Data: Small medical imaging datasets (hundreds rather than thousands of images) where transformers would overfit
(2) Computational Constraints: Deployment on edge devices, mobile platforms, or resource-limited clinical settings
(3) Real-Time Requirements: Applications requiring sub-second inference times without GPU acceleration
(4) Boundary Precision: Tasks where accurate delineation of fine structures is more important than classification accuracy
(5) Interpretability Needs: Clinical applications requiring explainable predictions and feature visualization
(6) Development Speed: Projects with limited time or expertise for implementing complex architectures
(7) Stable Texture Environments: When training and deployment data have consistent texture characteristics.

The architecture's longevity is not merely historical inertia but reflects genuine technical advantages for specific problem domains. While transformers and foundation models push the boundaries of what's possible with massive datasets and computational resources, U-Net continues to provide an optimal balance of simplicity, efficiency, and effectiveness for practical medical image segmentation tasks where data is limited, resources are constrained, and reliability is paramount (140).

### 4.4 Challenges in applying U-Net to different medical imaging modalities

The application of U-Net to different medical imaging modalities faces several challenges. Medical image datasets are scarce and difficult to obtain compared with ordinary computer vision datasets (106). This scarcity is due to privacy concerns, limited availability of annotated data, diversity of imaging modalities (e.g., x-ray, MRI, CT, and ultrasound), and the need for annotation by medical professionals (141). Improving image quality and standardizing imaging protocols across different modalities are also critical. Problems include variations in image acquisition from different medical devices, lack of uniform standards for annotation and CT/MRI machine performance, and inconsistencies in image quality, which affect model generalization. In addition, medical imaging requires extremely high accuracy for disease diagnosis. Related concerns include the difficulty of distinguishing boundaries between multiple cells and organs, the need for pixel- or voxel-level segmentation, and continuous parameter adjustment for achieving and maintaining accuracy. Furthermore, professionals lack confidence in applying DL model predictions to medical images. Specific problems include the poor interpretability of U-Net (106). In addition, gaining the trust and acceptance of expert physicians for clinical applications is difficult, and achieving interpretability while maintaining performance is a complex objective.

## 5 Applications for computer vision in real-world medical imaging

Revolutionizing healthcare diagnosis and treatment, computer vision has numerous real-world applications in medical imaging (94). In radiology and diagnostic imaging, computer vision algorithms help radiologists detect and classify abnormalities in x-rays, CT scans, and MRIs. For example, DL models can identify lung nodules in CXRs with high accuracy, aiding in the early detection of lung cancer (142). Computer vision is also used in digital pathology to analyze tissue samples and detect cancer cells (143). These systems can quickly process large volumes of slides, improving the efficiency and accuracy of cancer diagnosis. In addition, AI-powered systems are used to analyze retinal images to detect eye diseases, such as diabetic retinopathy, glaucoma, and age-related macular degeneration (AMD) (144–146). This technology enables early diagnosis and intervention, potentially preventing vision loss. Computer vision algorithms can also analyze skin lesions in photographs, helping dermatologists identify potential skin cancers and other dermatological conditions (147). In surgical planning and guidance, the 3D reconstruction of medical images helps surgeons plan complex procedures (148). During surgery, computer vision is used in augmented reality systems to overlay critical information on the surgeon's view (149). In cardiovascular imaging, echocardiograms and coronary CT angiography images are analyzed using AI algorithms to detect heart abnormalities and assess cardiovascular risk (150). In neuroimaging, computer vision techniques are used to analyze brain scans to help diagnose neurological disorders, such as Alzheimer's disease, multiple sclerosis, and brain tumors (80, 151–153).

AI-based systems can also detect and classify polyps in real time during colonoscopy, improving the accuracy of colorectal cancer screening (154). Computer-aided detection (CAD) systems assist radiologists in identifying potential breast cancer lesions on mammograms, increasing detection rates and reducing false negatives (155). AI algorithms are used in emergency medicine to analyze CT scans and detect critical conditions rapidly, such as intracranial hemorrhage, accelerating treatment in time-sensitive situations (156). These applications demonstrate the value of computer vision in enhancing medical imaging across multiple specialties, where it improves diagnostic accuracy, treatment planning, and patient outcomes.

## 6 Computer vision techniques for cancer detection

Computer vision techniques contribute substantially to cancer detection by improving the accuracy, efficiency, and accessibility of diagnostic procedures. These techniques use advanced image processing algorithms and ML to analyze various types of medical images, including x-rays, CT scans, MRIs, and histopathology slides (157). Computer vision algorithms can detect subtle abnormalities in medical images, enabling early cancer detection/diagnosis (4). This capability is critical for improving treatment outcomes and patient prognoses. AI-powered systems can analyze large volumes of medical imaging data with high accuracy, often outperforming traditional diagnostic methods (improved accuracy) (158). For example, HiDisc, developed by researchers at the University of Michigan, has demonstrated almost 88% accuracy in certain cases. CAD systems can rapidly analyze medical images, reducing the time required for diagnosis from days to minutes (improved efficiency) (159). Consequently, healthcare professionals can focus more on patient care. AI-powered systems also act as “second pairs of eyes” for clinicians, helping them identify potential cancerous lesions or tumors that may be missed during routine examinations. Computer vision models can apply the expertise of top oncologists to image analysis, making high-quality cancer detection more accessible in rural areas and developing countries (democratization of expertise) (4).

AI algorithms help detect lung nodules in CXRs and analyze mammograms for breast cancer screening (160). Regarding digital pathology, computer vision is used to analyze tissue samples and detect cancer cells in histopathology slides (143). Advanced techniques, such as transfer learning, ensemble learning, and vision transformers (ViTs), are used to integrate information from different imaging modalities, such as CT, MRI, and positron emission tomography scans, for comprehensive cancer detection (multimodal analysis) (4).

The ViT architecture involves several key formulas. Its key formula for self-attention is

$$s_{1}^{\;\;\prime }\;=\;softmax((q_{1}\cdot k_{1})/\surd d,(q_{1}\cdot k_{2})/\surd d,(q_{1}\cdot k_{3})/\surd d,\ldots )$$ (8)

where ***q_1_*** is the query vector; ***k_1_***, ***k_2_***, and ***k_3_*** are the key vectors; ***d*** is the dimension of the key vectors; and ***s_1_***′ is the attention weight vector.

The input image ***x*** ∈ RH × W × C is divided into patches, where H, W, and C are the height, width, and channels, respectively. These patches are then flattened and linearly embedded, with positional embeddings added to preserve spatial information. The resulting sequence is fed into a standard transformer encoder that applies multihead self-attention and feedforward layers. Final classification is performed using a softmax function on the output of the transformer encoder.

Although computer vision techniques offer considerable potential for cancer detection, challenges remain, including the need to improve the availability of high-quality labeled datasets, the diagnosis of rare cancers, and model explainability and generalization (4). Ongoing research aims to address these challenges and further enhance the role of computer vision in cancer detection and diagnosis.

## 7 Application of ML algorithms to medical images

### 7.1 U-Net with ML algorithms for image segmentation

U-Net was used to segment brain images in the Brain Tumor Segmentation challenge (161), where it helped identify and delineate brain tumors accurately in MRI scans. In addition, the architecture was used for liver image segmentation in the “siliver07” challenge for liver segmentation in CT scans (162), helping detect liver boundaries and identify potential lesions accurately. U-Net variants are also used for the semantic segmentation of OCT scans of the posterior segment of the eye. Specifically, they help identify different regions in OCT images of healthy eyes and eyes with conditions such as Stargardt's disease and AMD (163). Furthermore, U-Net is adopted to predict protein binding sites, which is critical for understanding protein–protein interactions and drug discovery (164), and to estimate fluorescent staining (in image-to-image translation), which is valuable in cellular imaging and analysis (165).

U-Net is implemented to analyze the micrographs of materials to characterize them and study their properties at microscopic scales (166). Pixelwise regression using U-Net is used for pansharpening, which combines high-resolution panchromatic and low-resolution multispectral satellite imagery (167). In addition, the U-Net architecture is used in diffusion models for iterative image denoising; in this application, it underlies modern image generation models, such as DALL-E, Midjourney, and Stable Diffusion (168). As for medical image reconstruction, U-Net variations have improved the quality and resolution of medical imaging techniques. 3D U-Net is used to learn dense volumetric segmentation from sparse annotation, which is particularly useful in 3D medical imaging (169). These applications demonstrate the versatility of U-Net in tackling different image analysis tasks in various domains, with each implementation being tailored to task-specific requirements.

### 7.2 You-only-look-once (YOLO) algorithms in detecting structures, anomalies, and instruments within medical images

YOLOv5 is used to detect and classify pressure ulcers in medical images, potentially improving patient outcomes and reducing healthcare costs (170). In CXR analysis, YOLOv5 and faster R-CNN were used to identify various abnormalities, with YOLOv5 demonstrating superior accuracy (email protected] 0.616 vs. faster R-CNN's 0.580) (171). YOLO algorithms are effective tools for tumor detection and localization in various medical imaging modalities (172). YOLOv8 enables real-time processing when applied to MRI images for brain tumor detection (172). YOLO models can identify tumors, lesions, and other clinically relevant medical objects in various imaging modalities (173). Likewise, YOLO can detect and localize anatomical structures, helping diagnose cardiovascular, neurological, and other conditions (170). YOLOv8 introduces several enhancements, including a refined spine network and neck portion (influenced by YOLOv7's efficient layer aggregation network design), the adoption of a decoupled head structure, a shift from an anchor-based approach to an anchor-free one, and improved data augmentation (172). Ultralytics' YOLOv11 further enhances medical imaging, particularly for tumor detection, by enabling radiologists to handle higher case volumes with consistent quality and improving real-time object detection and interpretation of complex images (174).

However, despite their successes, YOLO algorithms face certain challenges in medical imaging, including the need for large and balanced datasets, high computational requirements, limited flexibility with highly heterogeneous images, suboptimal sensitivity and precision for images such as MRIs, and possible inadequacy of standard loss functions for precise boundary localization in medical images (172, 175). Researchers are addressing these limitations by developing more flexible convolutional networks to adapt to complex image features, improving sensitivity and accuracy for specific medical imaging modalities, or designing specialized loss functions for better boundary localization in medical images (172). These ongoing improvements aim to improve the performance of YOLO algorithms in medical applications, particularly in handling images with complex backgrounds and unclear boundaries.

## 8 Active learning, reinforcement learning, and federated learning for medical images

### 8.1 Potential of active learning, reinforcement learning, and federated learning

Active learning, reinforcement learning, and federated learning provide innovative solutions to various challenges in medical imaging. Active learning is particularly useful for medical imaging because of the considerable cost and time consumption of data labeling (176). Active learning has been applied to the classification of CXR images for COVID-19 detection, significantly reducing the required labeling (177). For example, structure-and-boundary-consistency-based active learning was developed for medical image segmentation (178); open-set active learning was used for nucleus detection in medical images (179); and foundation-model-driven active barely supervised learning (FM-ABS) was adopted for 3D medical image segmentation (180).

Reinforcement learning, especially deep reinforcement learning, has several applications in medical imaging (181). It has been used to identify key anatomical points in medical images, detect and localize objects or lesions in medical scans, align medical images from different modalities or time points, determine optimal viewing planes in 3D imaging, optimize ML model parameters for medical imaging tasks, and segment and analyze surgical motion in medical procedures.

Federated learning is gaining traction in medical imaging because it enables collaborative model training while maintaining privacy (182, 183). Multiple medical institutions can collaboratively train models without sharing raw patient data. Federated MRI reconstruction techniques are used to improve image quality across multiple centers. Federated learning is used to segment tumors in medical images from multiple institutions. Additionally, Fed-CBT generates representative connectivity maps from multicenter brain imaging data, whereas FedFocus was developed for COVID-19 detection in CXRs across multiple healthcare centers. These applications demonstrate the potential of active, reinforcement, and federated learning to address critical challenges in medical imaging, including data scarcity, decision-making complexity, and privacy concerns (184).

### 8.2 Problems in medical imaging

ML and computer vision algorithms are used in medical imaging to solve broad-ranging problems and enhance diagnostic capabilities and patient care. These algorithms are primarily used for disease detection and diagnosis, image segmentation, early disease detection, image analysis and interpretation, surgical assistance, patient monitoring, workflow optimization, and multimodal analysis. AI systems can identify various conditions, such as tumors, lesions, and anatomical abnormalities, in medical images, such as x-rays, MRIs, CT scans, and ultrasound images (17, 29). For example, they can detect breast cancer in mammograms with higher accuracy (fewer false positives and false negatives) than human radiologists (185).

DL algorithms, particularly CNNs, accurately and efficiently segment medical images, helping isolate specific structures or regions of interest (17, 31). These algorithms recognize subtle patterns in medical images and patient records, enabling the early detection of diseases such as cancer, Alzheimer's, and cardiovascular disease, often before symptoms develop. AI models can automatically extract meaningful features from medical images for efficient, accurate interpretation. This entails disease classification, assessment of disease progression, and evaluation of treatment response (17). Computer vision algorithms can assist in surgical planning and guidance through a real-time analysis of medical images during surgery (186). These technologies enable the real-time monitoring of patient conditions, which is particularly useful for treating chronic conditions, postoperative recovery, and elderly care. By automating image analysis, these algorithms help streamline hospital workflows, allowing radiologists to prioritize urgent cases and reducing their manual workload. DL algorithms facilitate comprehensive analysis by integrating data from multiple imaging modalities, thus improving the overall diagnostic process. By addressing these issues, ML and computer vision algorithms in medical imaging improve diagnostic accuracy, accelerate clinical decision-making, and ultimately improve patient outcomes.

### 8.3 Performance comparison of ML and computer vision algorithms

As powerful tools for data analysis and image processing, ML and computer vision algorithms have become increasingly important in various fields. Performance insights by some tasks are summarized in Table 2 (187).

**Table 2:** Performance insights by task.

Tasks	Models	Performances
Classification tasks	ResNet-50	98.37% accuracy (chest x-ray)
	DeiT-Small	92.16% accuracy (brain tumor)
	EfficientNet-B0	81.84% accuracy (skin cancer)
	Fine-Tuned ResNet50	98.20% accuracy, 99.00% precision, 98.82% recall, 98.91% F1-score (COVID-19)
Segmentation tasks	DenseNet-121 U-Net	0.79–0.87 precision, 0.92–0.97 recall
	Diffusion-CSPAM-U-Net	84.4% DSC, 73.1% IoU
	Attention UNet	85.36% IoU, 91.49% Dice score
Detection tasks	YOLOv5-v8	95%–99.17% precision, 97.5% sensitivity, >95% mAP
	YOLO-NeuroBoost	99.48% mAP (brain tumors)
	YOLOv10	20 ms inference time (kidney stones)

In addition, the object detection algorithms perform differently. LinearSVC performs the best across all metrics, followed by SGDC and logistic regression (Table 2).

Image classification performance:

Accuracy=(TruePositives)+(TrueNegatives)/TotalSamples

CNNs typically outperform traditional ML algorithms in image classification. A study comparing different architectures showed accuracies of 95.2%, 93.7%, and 94.5% for ResNet-50, VGG-16, and Inception-v3, respectively.

Researchers found that adjacency-matrix (AM) diagrams consistently outperformed node–link (NL) diagrams in graph visualization tasks, specifically counting tasks, connectivity tasks, and performance degradation. The performance superiority of AM was more evident for larger graphs. NL accuracy decreased significantly for graphs with more than 30 nodes, but AM remained stable (188).

Different algorithms excel at varying tasks. CNNs are superior for image-related tasks, whereas support vector machines (SVMs) and random forests perform well on structured data. DL algorithms generally outperform traditional ML algorithms on large datasets but require more computational resources. Simpler models, such as decision trees, offer better interpretability, whereas complex models, such as neural networks, offer higher accuracy at the cost of interpretability. DL models typically require larger datasets to achieve optimal performance compared with traditional ML algorithms. Feature scaling and data preprocessing substantially affect the performance of many algorithms, especially KNNs and SVMs. In graph-related tasks, AM diagrams outperform NL diagrams, especially for larger and denser graphs. Thus, the choice of algorithm depends on the task, dataset size, available computational resources, and interpretability requirement. As the field evolves, hybrid approaches combining traditional ML and DL techniques are emerging, aiming to leverage the strengths of both paradigms (189).

## 9 Critical thoughts and research questions for computer vision in medical imaging

Computer vision in medical imaging has revolutionized disease detection, diagnosis, and image interpretation. Computer vision algorithms can remarkably detect disease in medical images. AI systems can identify lung nodules in chest CT scans at sensitivities comparable to those of experienced radiologists (190). DL algorithms can detect breast cancer in mammograms with greater accuracy (fewer false positives and false negatives) than human radiologists (191). AI systems can detect diabetic retinopathy in retinal images with high accuracy (90.3% sensitivity and 98.1% specificity), enabling early intervention (192).

Computer vision is improving medical image analysis through the automated analysis of large volumes of medical images, reducing radiologists' workload and increasing efficiency; accurate interpretation of x-rays, MRIs, and CT scans, which helps identify abnormalities and accelerates diagnosis; and advanced techniques, such as image segmentation, object detection, disease classification, and image reconstruction (17, 141). However, the following research questions remain: (i) Data quality and quantity: How can we ensure the availability of high-quality, diverse, well-annotated medical imaging datasets for training robust AI models? (ii) Interpretability: How can we develop explainable AI models that have a transparent decision-making process, which is critical for clinical confidence and adoption? (iii) Generalization: How can we develop AI models that perform consistently across different patient populations, imaging devices, and healthcare settings? (iv) Integration into clinical workflows: How can computer vision tools be seamlessly integrated into existing clinical workflows to improve efficiency without compromising patient care? (v) Ethical and legal considerations: What are the ethical implications of using AI in medical imaging, and how can we address liability and patient privacy problems? (vi) Multimodal integration: How can we combine computer vision with other data sources (e.g., patient history and genomics) for more comprehensive and accurate diagnoses? (vii) Real-time analysis: How can we develop computer vision algorithms capable of real-time analysis for time-critical applications, such as stroke detection and surgical assistance? (viii) Validation and clinical trials: How can we rigorously validate computer vision algorithms in clinical settings and ensure their safety and efficacy? (ix) Continuous learning: How can we design AI systems that can adapt and improve over time as they encounter new data and clinical scenarios? (x) Resource optimization: How can computer vision technologies be optimized to run efficiently on limited computational resources and thus be accessible in different healthcare settings? (193, 194). These research questions and critical considerations should be addressed to advance the field of computer vision in medical imaging and realize its full potential to improve patient care and outcomes.

**Figure 3:**
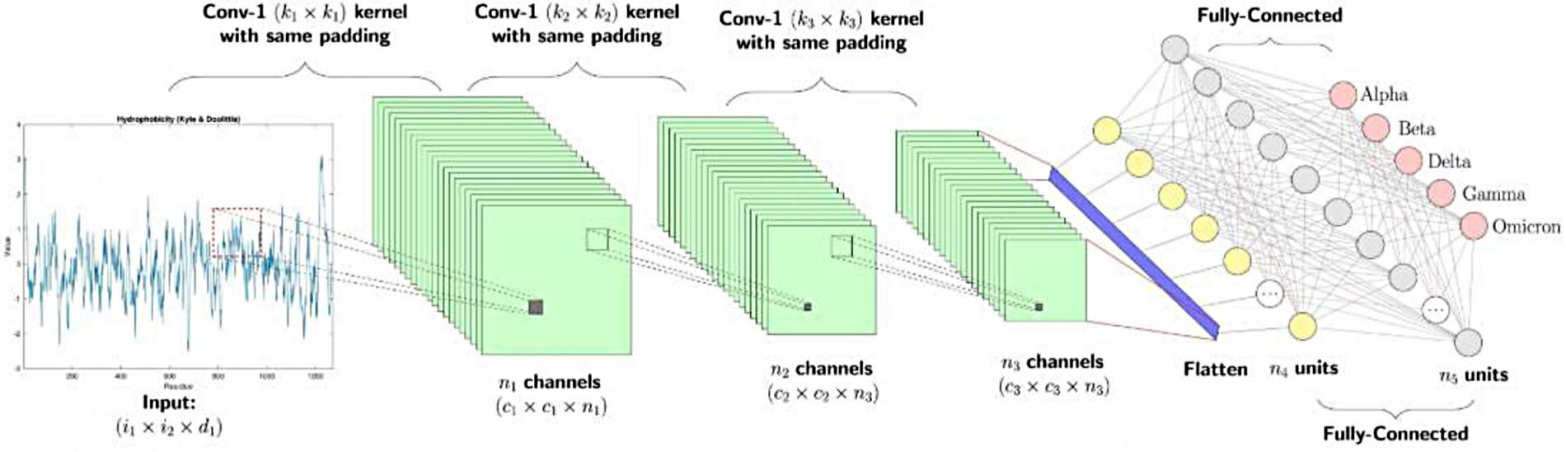
A deep learning model processing time-series data. CNN architectures are structured pipelines of layers designed to analyze visual data by using a feature extractor (convolutional, pooling, and activation layers) followed by a classifier (fully connected layers). Key components include convolutional layers for feature extraction, pooling layers to reduce spatial dimensions, and fully connected layers to perform the final classification.

**Figure 4:**
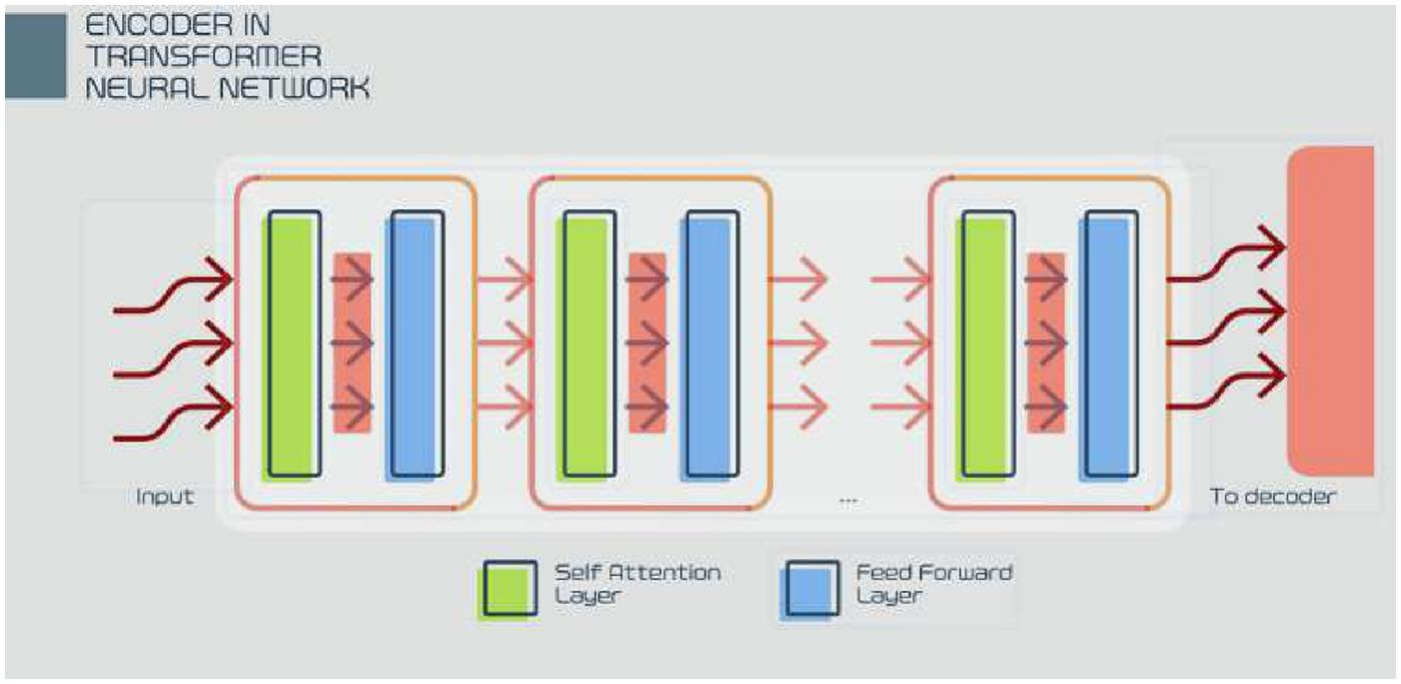
The architecture of the encoder in a transformer neural network. A transformer is a deep learning model that adopts the mechanism of self-attention, differently weighing the significance of each part of the input data. The encoder's job is to process the input data, such as a sentence in a source language, and convert it into a high-dimensional representation.

Deep learning has revolutionized medical imaging by enabling automated, faster, and often more accurate analysis of various imaging modalities, such as x-rays, CT scans, MRIs, and ultrasound (17, 30, 195). This success is primarily due to the ability of deep learning models, especially CNNs, to automatically learn complex, hierarchical features directly from raw image data, eliminating the need for manual feature engineering. The most used deep learning architectures and techniques in medical imaging include CNNs, U-Net, Recurrent Neural Networks (RNNs)/Long Short-Term Memory (LSTMs), Generative Adversarial Networks (GANs), and Transformers (196). The foundational model for most computer vision tasks, CNNs use convolutional layers to extract spatial hierarchies of features (like edges, textures, and shapes) from images. They are the backbone for classification, detection, and segmentation tasks (Figure 3). A highly popular variant of CNNs, U-Net, particularly for image segmentation features a symmetric encoder-decoder architecture with skip connections that allow high-resolution feature maps from the encoder path to be combined with the upsampled output of the decoder (123). This combination helps produce very precise segmentation masks (Figure 4). While less common than CNNs for 2D images, RNNs are useful for tasks involving sequential data, such as analyzing adjacent slices in 3D scans (like CT or MRI) or tracking disease progression over time (4). These GANs models consist of a generator and a discriminator network that compete, allowing them to create synthetic, realistic-looking medical images (197). GANs are used for data augmentation (especially for rare diseases), image denoising, and reconstruction (e.g., low-dose CT reconstruction) (198). Also, architectures (adapted for vision) of Transformer, initially successful in natural language processing, are increasingly being used in medical imaging for tasks requiring a global understanding of the image content (199).

Deep learning methods are applied across the entire medical imaging pipeline, including i) image classification: identifying the presence of a disease (e.g., classifying a mammogram as benign or malignant) or categorizing a condition, ii) object detection and localization: identifying the precise location of abnormalities, lesions, or organs within an image using bounding boxes (e.g., detecting lung nodules on a chest x-ray), iii) image segmentation: assigning a label to every pixel, effectively outlining the boundaries of tumors, organs, or other structures (200). This is crucial for precise treatment planning, iv) computer-aided diagnosis (CAD): Providing a second, automated opinion to radiologists to help flag potentially missed findings, increasing efficiency and reducing error rates, and v) image reconstruction and enhancemen: low-dose CT/denoising: removing noise from images acquired with lower radiation doses and super-resolution: generating high-resolution images from lower-resolution scans (especially for MRI) (201). Despite their success, the application of deep learning in a clinical setting still faces several challenges, such as data scarcity and annotation, interpretability (Explainable AI - XAI), and generalization (202). Medical images are difficult and expensive to label accurately by experts, and datasets for specific rare diseases are often small (203). Also, the “black box” nature of deep learning makes it difficult for clinicians to understand why a model made a specific prediction, which is a significant barrier to clinical trust and adoption (204). Models often perform poorly when tested on data from different hospitals, scanners, or patient populations than those they were trained on. Therefore, future research focuses on addressing these challenges through transfer learning and foundation models; leveraging models pre-trained on large, general datasets and fine-tuning them on smaller medical datasets, self-supervised and semi-supervised learning; training models effectively with limited labeled data, and federated learning; training models across multiple institutions without sharing patient data, helping to improve generalization while maintaining privacy.

## 10 Critical synthesis in the field of computer vision in medical imaging

Computer vision has become a transformative force in medical imaging, with rapid advancements in deep learning reshaping diagnostic workflows and clinical decision-making (29). This synthesis examines the critical developments, persistent challenges, and emerging paradigms in this rapidly evolving field.

### 10.1 The paradigm shift to generative and unified models

A fundamental evolution is occurring in medical imaging AI. Medical Vision Generalist (MVG) represents the first foundation model capable of handling various medical imaging tasks such as cross-modal synthesis, image segmentation, denoising, and inpainting within a unified framework. This marks a departure from task-specific models toward versatile, adaptable systems that can generalize across imaging modalities. Recent developments focus on methods for image-to-image translation, image synthesis, biophysical modeling, super-resolution and image segmentation, demonstrating how synthesis techniques are becoming central to addressing data scarcity and enhancing image quality (205). The integration of generative AI enables clinicians to overcome traditional limitations like equipment costs and accessibility barriers, as evidenced by work on generating 3D OCT images from 2D fundus photographs (206).

### 10.2 The interpretability imperative

Despite impressive accuracy gains, the black-box nature of deep learning models remains the most significant barrier to clinical adoption. State-of-the-art deep learning models have achieved human-level accuracy on classification of different types of medical data, yet these models are hardly adopted in clinical workflows, mainly due to their lack of interpretability (207). Five core elements define interpretability in medical imaging machine learning: localization, visual recognizability, physical attribution, model transparency, and actionability (24). The field is increasingly recognizing that inherently interpretable models may be more valuable than *post hoc* explanation methods. Techniques like attention mechanisms, saliency mapping, and gradient-based visualization are evolving to provide clinically meaningful explanations that radiologists can trust and act upon (208). Key challenges include data availability, interpretability, overfitting, and computational requirements, underscoring that technical performance alone is insufficient for real-world deployment.

### 10.3 Privacy-preserving collaborative learning

The tension between data-hungry deep learning models and stringent privacy regulations has catalyzed federated learning research (209). Federated learning offers a privacy-preserving solution by enabling collaborative model training across institutions without sharing sensitive data, with model parameters exchanged between participating sites (210). However, federated learning faces its own challenges. Sensitive information can still be inferred from shared model parameters, and post deployment data distribution shifts can degrade model performance, making uncertainty quantification essential. Emerging solutions combine differential privacy, secure multi-party computation, and homomorphic encryption to strengthen privacy guarantees while maintaining model performance. Differentially private federated learning has achieved strong privacy bounds with performance comparable to centralized training, demonstrating viability for real-world medical applications (211).

### 10.4 The data heterogeneity challenge

Medical imaging presents unique challenges that confront deep learning approaches, with billions of medical imaging studies conducted per year worldwide, and this number is growing (194). The heterogeneity of medical data—across institutions, imaging protocols, patient populations, and equipment—creates significant generalization challenges. Challenges such as data variability, interpretability, and generalization across different patient populations and imaging modalities need to be addressed to ensure reliable and effective medical image analysis. Hybrid approaches that integrate traditional computer vision techniques with deep learning, alongside multi-modal learning strategies, are emerging as promising solutions (212).

### 10.5 Uncertainty quantification: from prediction to confidence

Clinical decision-making requires not just predictions but confidence estimates. In federated learning contexts, uncertainty quantification becomes particularly challenging due to data heterogeneity across participating sites. Methods including Bayesian approaches, ensemble techniques, and conformal prediction are being developed to provide clinicians with reliable uncertainty estimates that can guide treatment decisions (213).

### 10.6 Architectural innovation and efficiency

The field has progressed beyond basic CNNs to sophisticated architectures. U-Net and its variants remain dominant for segmentation tasks, while Vision Transformers (ViTs) are increasingly adopted for capturing long-range dependencies in medical images (137). Deep learning-based models achieve significant increases in segmentation, classification, and anomaly detection accuracies across various medical imaging modalities (214). Attention mechanisms enable models to focus on clinically relevant regions, while transfer learning and domain adaptation help overcome limited annotated data (215). The challenge remains balancing model complexity with computational efficiency, particularly for deployment in resource-constrained clinical settings.

Several critical gaps persist evaluation standards, clinical integration, robustness and fairness, and regulatory frameworks. The field lacks standardized benchmarks for evaluating interpretability, uncertainty quantification, and fairness across diverse patient populations. Despite technical advances, seamless integration with existing clinical workflows and electronic health record systems remains incomplete. Models trained on data from specific institutions often fail to generalize across diverse demographics, raising concerns about algorithmic bias and healthcare equity. The rapid pace of AI development has outpaced regulatory guidance, creating uncertainty around clinical validation and FDA approval pathways.

### 10.7 Toward trustworthy medical AI

The evolution of computer vision in medical imaging reflects maturation from purely accuracy-focused models toward systems that prioritize trustworthiness, privacy, and clinical utility (216). The convergence of foundation models, privacy-preserving techniques, and explainable AI represents a holistic approach to addressing the field's fundamental challenges. Success will require continued interdisciplinary collaboration between computer scientists, radiologists, ethicists, and policymakers. The goal is not simply to replicate human performance but to create AI systems that augment clinical expertise, reduce diagnostic errors, and ultimately improve patient outcomes while maintaining the highest standards of privacy and interpretability. The field stands at an inflection point where technical capability increasingly meets clinical need but realizing this potential demands addressing the critical challenges of interpretability, privacy, heterogeneity, and real-world generalization with the same rigor applied to achieving high accuracy metrics.

## 11 Quantitative comparisons, performance metrics, and systematic evaluations in computer vision for medical imaging

### 11.1 CNN vs. vision transformer performance comparisons

A systematic review analyzing 36 studies indicates that transformer-based models, particularly Vision Transformers, exhibit significant potential in diverse medical imaging tasks, showcasing superior performance when contrasted with conventional CNN models (217). As for task-specific performance, across three medical imaging tasks - chest x-ray pneumonia detection, brain tumor classification, and skin cancer detection - results demonstrate task-specific model advantages: ResNet-50 achieved 98.37% accuracy on chest x-ray classification, DeiT-Small excelled at brain tumor detection with 92.16% accuracy, and EfficientNet-B0 led skin cancer classification at 81.84% accuracy (Table 2) (218). Further, as for hybrid architecture advantages, a systematic review of 28 articles on hybrid ViT-CNN architecture identified that integrating ViT and CNN can mitigate the limitations of each architecture, offering comprehensive solutions that combine global context understanding with precise local feature extraction (219). The CSPDarknet53 backbone is the fastest with a decent receptive field, while EfficientNet-B3 has the largest receptive field but the lowest FPS, illustrating the fundamental trade-off between receptive field size and inference speed.

### 11.2 Benchmark datasets and evaluation frameworks

MedSegBench encompasses 35 datasets with over 60,000 images from ultrasound, MRI, and x-ray modalities, evaluating U-Net architectures with various encoder networks including ResNets, EfficientNet, and DenseNet, with DenseNet-121 consistently demonstrating strong performance across numerous datasets, achieving precision scores such as 0.794 in BusiMSB, 0.870 in ChuahMSB, and 0.801 in Dca1MSB (220). As for performance across encoders, DenseNet-121 emerged as a top performer for recall, obtaining the highest recall in datasets such as Bbbbc010MSB (0.920) and WbcMSB (0.970), while EfficientNet performed well in recall metrics, particularly in datasets like DynamicNuclearMSB (0.966) and USforKidneyMSB (0.982). Furthermore, MedMNIST v2 provides a collection of standardized biomedical images including 12 datasets for 2D and 6 datasets for 3D, consisting of 708,069 2D images and 9,998 3D images in total, with benchmarking showing that Google AutoML Vision performs well in general, though ResNet-18 and ResNet-50 can outperform it on certain datasets (221).

### 11.3 Imagenet performance metrics

Quantitative comparisons provide the essential metrics needed to evaluate and select computer vision algorithms for specific applications. ImageNet remains the gold standard benchmark for image classification. Top-1 error rate measures whether the top predicted label matches the ground truth, while Top-5 error rate checks if the correct label is among the top five predictions. The evolution of architectures shows dramatic improvements such as AlexNet (2012), 15.4% top-5 error rate, marking the deep learning revolution, VGG-16/19 (2014), 7.3% top-5 error rate, 138 million parameters, ResNet (2015), ResNet achieved a top-5 error rate as low as 3.57%, which refreshed the record of precision of CNNs on ImageNet, EfficientNet-B7, Achieved a top-one accuracy of 84.3 percent on ImageNet, and Modern models (2024–2025), CoCa achieves 91.0% top-1 accuracy on ImageNet after fine-tuning, but requires 2.1B parameters.

EfficientNet-B1 is 7.6× smaller and 5.7× faster than ResNet-152, demonstrating how architectural innovation can dramatically improve the accuracy-to-efficiency trade-off (222). ResNet-50 is faster than VGG-16 and more accurate than VGG-19, while ResNet-101 is about the same speed as VGG-19 but much more accurate than VGG-16 (223). The computational differences are stark: VGG-16 requires roughly 138 million parameters with ResNet having 25.5 million parameters, though parameter count alone doesn't determine speed. VGG's early convolutional layers on full-resolution images create massive computational costs, while ResNet's early downsampling strategy dramatically reduces FLOPs.

### 11.4 Current state-of-the-art (2025)

EfficientNet provides the best accuracy-to-parameter ratio, with EfficientNet-B0 achieving 77.1% accuracy with only 5.3M parameters, while ConvNeXt V2 offers a strong balance with the Tiny variant achieving 83.0% accuracy using 28.6M parameters. For comparative performance, in general, Faster R-CNN is more accurate while R-FCN and SSD are faster, with Faster R-CNN using Inception ResNet with 300 proposals giving the highest accuracy at 1 FPS for all tested cases (224).

### 11.5 U-Net vs. mask R-CNN performance

Quantitative comparisons in medical imaging reveal task-specific performance differences, such as panoramic radiograph segmentation, lung segmentation in CT, pancreas segmentation, cardiac MRI segmentation, and brain tumor segmentation (225). Multi-Label U-Net achieved a dice coefficient of 0.96 and an IoU score of 0.97, while Mask R-CNN attained a dice coefficient of 0.87 and an IoU score of 0.74, with Mask R-CNN showing accuracy, precision, recall, and F1-score values of 95%, 85.6%, 88.2%, and 86.6% respectively (226). Mask R-CNN coupled with a K-means kernel delivered the best segmentation results, achieving an accuracy of 97.68 ± 3.42% with an average runtime of 11.2 s (32). The maximum Dice Similarity Coefficients value for automatic pancreas segmentation techniques is only around 85% due to the complex structure of the pancreas, demonstrating that certain anatomical structures remain challenging even for state-of-the-art models (227). U-Net achieved a mean DSC that reached 97.9% and a mean Hausdorff Distance that reached 5.318 mm for cardiac MRI segmentation (228). Hybrid CNN models U-SegNet, Res-SegNet, and Seg-U-Net achieved average accuracies of 91.6%, 93.3%, and 93.1% respectively on the BraTS dataset (32).

### 11.6 Segmentation metrics - dice coefficient and IoU

As for U-Net performance metrics, for lung area segmentation in medical images using U-Net, the Dice coefficient increased from 0.5 to 0.9, while the IOU value stabilized at 0.9, representing the model's efficiency in proper segmentation with minimal overfitting as shown by the loss metrics' consistent reduction (229). In prostate segmentation using U-Net with nine different loss functions, Focal Tversky loss function achieved the highest average DSC scores for the whole gland at 0.74 ± 0.09, while models using IoU, Dice, Tversky and weighted BCE + Dice loss functions obtained similar DSC scores ranging from 0.71 to 0.73 (134). In Advanced U-Net Variants, the diffusion-CSPAM-U-Net model for brain metastases segmentation achieved internal validation results with DSC of 84.4% ± 12.8%, IoU of 73.1% ± 12.5%, accuracy of 97.2% ± 9.6%, sensitivity of 83.8% ± 11.3%, and specificity of 97.2% ± 13.8% (230). F-measure based metrics like Dice Similarity Coefficient and Intersection-over-Union are highly popular and recommended in medical image segmentation, with the difference being that IoU penalizes under- and over-segmentation more than DSC, and these metrics focus on true positive classification without true negative inclusion, providing better performance representation in medical contexts (231).

### 11.7 YOLO performance in medical imaging

The review highlights impressive performance of YOLO models, particularly from YOLOv5 to YOLOv8, in achieving high precision up to 99.17%, sensitivity up to 97.5%, and mAP exceeding 95% in tasks such as lung nodule, breast cancer, and polyp detection, demonstrating significant potential for early disease detection and real-time clinical applications (232). Across 123 peer-reviewed papers published between 2018 and 2024, mAP scores exceeding 85% were commonly achieved in breast cancer and pulmonary nodule detection tasks, while lightweight versions such as YOLOv5s maintained detection speed above 50 FPS in surgical environments (233). The YOLO-NeuroBoost model for brain tumor detection in MRI images achieved mAP scores of 99.48 on the Br35H dataset and 97.71 on the open-source Roboflow dataset, indicating high accuracy and efficiency in detecting brain tumors (172). In kidney stone detection comparing YOLOv8 and YOLOv10, YOLOv10 demonstrated faster inference at approximately 20 milliseconds compared to YOLOv8's 30 milliseconds, largely due to its NMS-free architecture, making it better suited for real-time detection where both speed and accuracy are critical.

YOLOv4 achieves 43.5% mAP at a real-time speed of approximately 65 FPS on the Tesla V100 GPU, running twice as fast as EfficientDet with comparable performance, and improving YOLOv3's mAP and FPS by 10% and 12% respectively on the MS COCO dataset.

In comparative analysis for skin disease classification, YOLOv5n achieved the highest accuracy of 0.942 with a training time of 1.204 h, while ResNet50 showed accuracy of 0.571 in detecting chickenpox, 0.666 in measles, 0.803 in monkeypox, and 0.864 in normal cases. EfficientNet_b2 demonstrated the highest accuracy among EfficientNet models in this application. As critical insights from quantitative comparisons, the quantitative data reveals several important patterns. Modern architectures like EfficientNet and ConvNeXt achieve similar or better accuracy than older models with dramatically fewer parameters. The speed-accuracy trade-off remains fundamental—YOLO excels in real-time detection while Faster R-CNN provides superior accuracy at lower speeds (234). For medical imaging, U-Net consistently outperforms Mask R-CNN for semantic segmentation tasks, while Mask R-CNN excels when instance-level separation is required (111). Task-specific factors like anatomical complexity significantly impact achievable accuracy regardless of architecture choice (235). The evolution from AlexNet's 84.6% accuracy to modern models exceeding 91% demonstrates the field's rapid progress, though improvements are increasingly marginal as models approach theoretical limits on benchmark datasets (236).

### 11.8 Few-shot learning performance

A systematic review analyzing relevant articles found that few-shot learning techniques can reduce data scarcity issues and enhance medical image analysis speed and robustness, with meta-learning being a popular choice because it can adapt to new tasks with few labelled samples (237). Experiments on four few-shot segmentation tasks show that Interactive Few-Shot Learning approach outperforms state-of-the-art methods by more than 20% in the DSC metric, with the interactive optimization algorithm contributing approximately 10% DSC improvement for few-shot segmentation models (238).

### 11.9 Foundation model benchmarking

A novel dataset and benchmark for foundation model adaptation collected five sets of medical imaging data from multiple institutes targeting real-world clinical tasks, examining generalizability across varied data modalities, image sizes, data sample numbers, and classification tasks including multi-class, multi-label, and regression (239). Also, evaluation of foundation models on patch-level pathology tasks revealed that pathology image pre-trained foundation models consistently outperformed those based on common images across all datasets, with no consistent winner across all benchmark datasets, emphasizing the importance of measuring performance over a diverse set of downstream tasks (240).

### 11.10 Standard evaluation metrics definitions

Standard metrics reported in medical imaging studies include accuracy, precision (ranging from 0.86–0.91 in validation studies), recall (ranging from 0.83 in studies), and F1 scores (ranging from 0.84–0.87), though AUROC and AUPRC cannot be calculated without access to the model or confusion matrix entries (241). Also, as for metric dependencies, accuracy, positive predictive value, negative predictive value, AUCPRC, and F1 score are functions of prevalence, while AUCROC, sensitivity, specificity, false-positive rate, and false-negative rate are not dependent on prevalence, making AUCPRC particularly suitable for scenarios with class imbalance (242).

### 11.11 Comparative architecture performance

In hip fracture detection using pelvic radiographs, YOLOv5 achieved 92.66% accuracy on regular images and 88.89% on CLAHE-enhanced images, while classifier models including MobileNetV2, Xception, and InceptionResNetV2 achieved accuracies between 94.66% and 97.67%, significantly outperforming clinicians' mean accuracy of 84.53% (243). For intracranial aneurysm detection, ResNet reached sensitivity of 91% and 93% for internal and external test datasets respectively, while CNN classifiers achieved detection of 94.2% of aneurysms with 2.9 false positives per case (Table 2) (244).

### 11.12 Dataset quality and duplication and bias and fairness metrics

The proliferation of duplicate datasets poses significant impediments to reproducibility, with the ISIC skin lesion dataset having 27 versions on HuggingFace and 640 datasets on Kaggle totaling 2.35 TB of data compared to the original 38 GB, with many lacking original sources or license information. Current study reveals significant correlation between each model's accuracy in making demographic predictions and the size of its fairness gap, suggesting models may be using demographic categorizations as shortcuts to make disease predictions (245). This comprehensive quantitative overview demonstrates that model selection should be task-specific, with transformers generally outperforming CNNs for tasks requiring global context, while hybrid approaches and properly optimized CNNs remain competitive for many applications (246). Performance evaluation should use multiple complementary metrics appropriate to the clinical context and class balance.

## 12 New taxonomy or experimental validation, fresh perspectives, techniques, or frameworks for models and problems in the field of computer vision in medical imaging

### 12.1 Vision transformers (ViTs) - beyond traditional CNNs

As for hierarchical multi-scale approaches, recent research introduces hierarchical multi-scale Vision Transformer frameworks that incorporate innovative attention methodologies, using multi-resolution patch embedding strategies (8 × 8, 16 × 16, and 32 × 32 patches) for feature extraction across different spatial scales, achieving 35% reduction in training duration compared to conventional ViT implementations (247). Systematic reviews indicate that transformer-based models, particularly ViTs, exhibit significant potential in diverse medical imaging tasks, showcasing superior performance when contrasted with conventional CNN models, with pre-training being particularly important for transformer applications (217). There are some important innovations, RanMerFormer implements a randomized approach to combining visual tokens, substantially reducing computational demands while classifying brain tumors. Lesion-Centered Vision Transformers integrate lesion-focused MRI preprocessing with adaptive token merging for stroke outcome. Swin Transformers with linear complexity characteristics for processing MRI data prediction (248).

### 12.2 Foundation models - the paradigm shift

In Vision-Language Foundation Models (VLFMs), foundation models in the medical domain address critical challenges by combining information from various medical imaging modalities with textual data from radiology reports and clinical notes, enabling development of tools that streamline diagnostic workflows, enhance accuracy, and enable robust decision-making (249). Currently, there are some novel capabilities. NVIDIA's Clara NV-Reason-CXR-3B model generates detailed thought processes for chest x-ray analysis by capturing radiologist thought processes through voice recordings, with a two-stage training pipeline combining supervised fine-tuning with gradient reinforcement policy optimization. Zero-shot and few-shot performance across multiple downstream tasks. Foundation models exhibit remarkable contextual understanding and generalization capabilities, with active research focusing on developing versatile artificial intelligence solutions for real-world healthcare applications (250). Also, as challenges identified, studies investigating algorithmic fairness of vision-language foundation models reveal that compared to board-certified radiologists, these models consistently underdiagnose marginalized groups, with even higher rates in intersectional subgroups such as Black female patients (251).

### 12.3 Addressing data scarcity

Multi-task learning enables simultaneous training of a single model that generalizes across multiple tasks, taking advantage of many small- and medium-sized datasets in biomedical imaging by efficiently utilizing different label types and data sources to pretrain image representations applicable to all tasks (20). Physics-Informed Machine Learning (PIML) incorporates governing physical laws such as partial differential equations, boundary conditions, and conservation principles, guiding the learning process toward physically plausible and interpretable outcomes, reducing the need for large datasets while enhancing interpretability. In diffusion models for synthesis, conditional latent diffusion model-based medical image enhancement networks incorporate multi-attention modules and Rotary Position Embedding to effectively capture positional information, with findings indicating generated images can enhance performance of downstream classification tasks, providing effective solutions to scarcity of medical image training data (252). There are some critical approaches: GANs for data augmentation (though diffusion models are increasingly preferred), self-supervised learning on large unlabeled datasets, synthetic data generation with anatomical control, and cross-domain transfer learning (253).

### 12.4 Explainable AI (XAI) - building trust

As for novel XAI frameworks, recent work proposes explainable AI methods specifically designed for medical image analysis, integrating statistical, visual, and rule-based explanations to improve transparency in deep learning models, using decision trees and RuleFit to extract human-readable rules while providing statistical feature map overlay visualizations (25). Also, there are some important techniques: i) Grad-CAM and LIME for visual saliency maps (254), ii) attention-based saliency maps improve interpretability of pneumothorax classification, with studies combining saliency-based heatmaps with clinical decision tasks and validating their plausibility through qualitative feedback from radiologists (255), iii) concept-based explanations using activation vectors (256), and iv) counterfactual explanations showing how image changes would alter predictions. As critical insight, from a human-centered design perspective, transparency is not a property of the ML model but an affordance - a relationship between algorithm and users, making prototyping and user evaluations critical to attaining solutions that afford transparency (257).

In addressing bias and fairness, the PROBAST-AI tool is under development specifically for evaluating ML studies, while reporting guidelines such as FUTURE-AI and TRIPOD-AI assist authors in reporting studies according to the Fairness principle, promoting identification of bias sources (258). Surprisingly, models with less encoding of demographic attributes are often most “globally optimal”, exhibiting better fairness during model evaluation in new test environments, though correcting shortcuts algorithmically effectively addresses fairness gaps within the original data distribution (245). Current study reveals significant correlation between each model's accuracy in making demographic predictions and the size of its fairness gap, suggesting models may be using demographic categorizations as shortcuts to make disease predictions. As mitigation strategies, it suggested i) subgroup robustness optimization, ii) group adversarial approaches to remove demographic information, iii) diverse dataset curation across demographics, and iv) regular fairness audits across deployment contexts (259).

### 12.5 Novel architecture and frameworks

**Figure 5:**
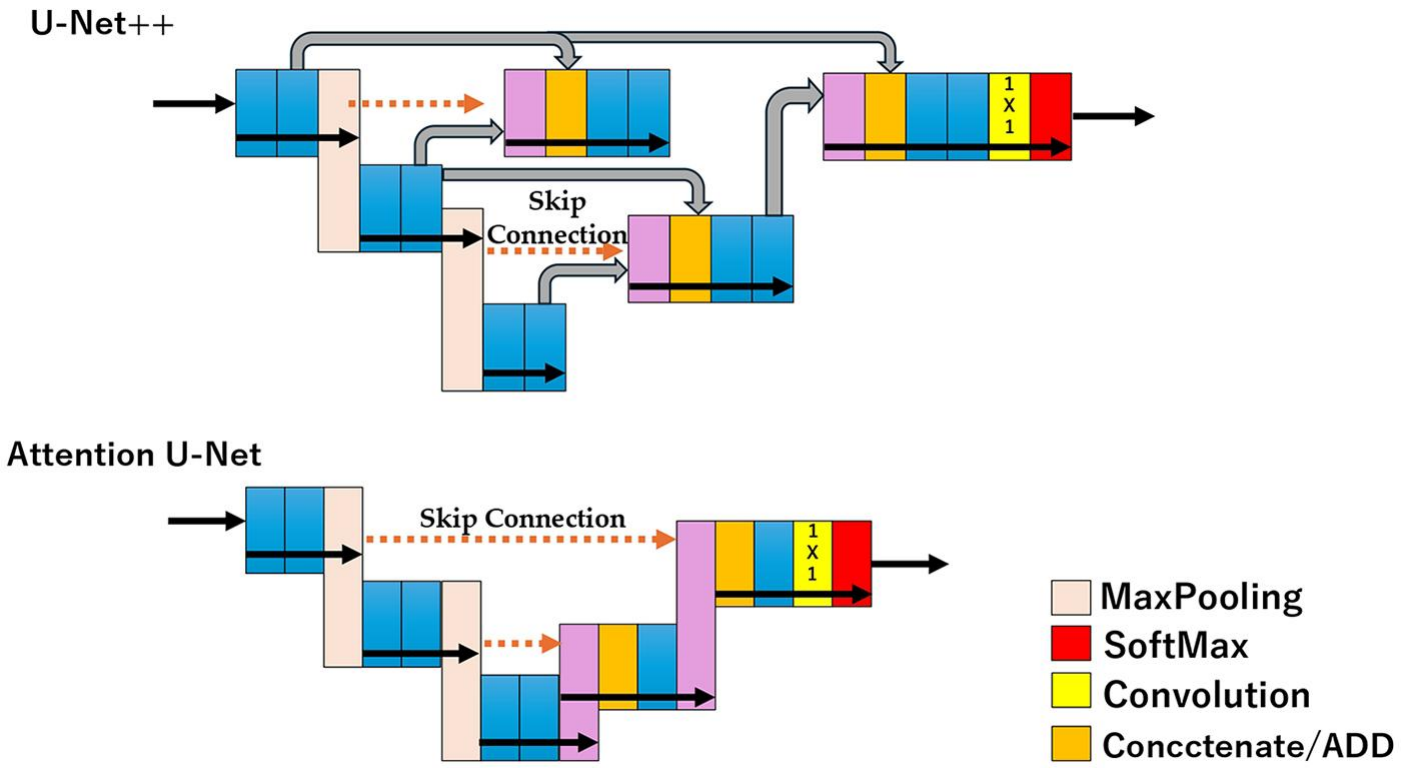
The U-Net++ and attention U-Net architectures are advanced variants of the original U-Net, designed to improve performance in image segmentation tasks, particularly in medical imaging. U-Net++ aims to improve the standard U-Net by addressing the semantic gap between the encoder and decoder feature maps connected by skip connections. It achieves this through a nested, densely connected structure. Instead of a single U-shaped path, U-Net++ incorporates multiple U-Net-like structures nested within each other. This creates pathways of varying depths (Nested U-Net Structure). Attention U-Net enhances the U-Net architecture by integrating attention mechanisms, specifically Attention Gates (AGs), into the skip connections. AGs are placed in the skip connections between the encoder and decoder. They learn to suppress irrelevant regions in the input image and highlight salient features relevant to the target structure.

In U-Net evolution, U-Net++ represents a nested U-Net architecture for medical image segmentation, while multi-scale attention U-Net with EfficientNet-B4 encoder enhances MRI analysis (117). CNN, Deep Learning algorithm that takes as an image or a multivariate time series, can successfully capture the spatial and temporal patterns through the application of trainable filters, and assigns importance to these patterns using trainable weights (Figure 5). The preprocessing required in CNN is much lower than compared to other classification algorithms. While in many methods filters are hand-engineered, CNN can learn these filters. As diffusion models for medical imaging, there are applications in image-to-image translation, reconstruction, registration, and anomaly detection and atomically controllable generation following multi-class segmentation masks. Diffusion probabilistic models synthesize high-quality medical data for MRI and CT, with radiologists evaluating synthetic images on realistic appearance, anatomical correctness, and consistency between slices (260). Also, as hybrid approaches, there are multi-modal fusion networks integrating imaging with clinical data and cross-attention mechanisms for modality integration. CNN-Transformer hybrids combining local and global feature extraction (261).

Transformer-based models have fundamentally reshaped computer vision, moving beyond the convolutional paradigm that dominated for decades (262). This analysis explores Vision Transformers (ViTs), Segment Anything Model (SAM), and Swin-U-Net architectures, examining their mechanisms, performance characteristics, and transformative impact on the field (263).

### 12.6 Architecture and applications of ViTs

#### 12.6.1 Fundamental architecture of ViTs

Vision Transformers revolutionized computer vision by adapting the transformer architecture from NLP to image processing. ViT builds upon the transformer architecture using self-attention mechanisms to model global relationships across image patches (264). The architecture divides images into fixed-size patches (typically 16 × 16 pixels), flattens them into vectors, and processes them through transformer encoder blocks. The standard ViT-Base configuration uses 768-dimensional embeddings, 12 transformer layers, 12 attention heads, and approximately 86 million parameters. This design enables ViT to capture global dependencies from the earliest layers—a stark contrast to CNNs that build hierarchical features progressively (265).

#### 12.6.2 Performance and scaling characteristics of ViTs

ViT architecture designs have considerable impact on out-of-distribution generalization, though in-distribution accuracy might not be a very good indicator of OoD accuracy. These finding challenges conventional wisdom about model evaluation and highlight the importance of robust metrics beyond standard benchmarks. When properly scaled, ViTs achieve remarkable results. ViT-22B, currently the largest vision transformer at 22 billion parameters, advances state-of-the-art on several benchmarks and shows increased similarities to human visual object recognition. This massive scale was achieved through architectural innovations including parallel layers (reducing training time by 15%) and omitting biases in certain projections (increasing utilization by 3%).

#### 12.6.3 Data efficiency and training requirements of ViTs

ViTs typically require large amounts of training data to achieve their best performance, while CNNs can perform well even with smaller datasets (217). This data hunger stems from ViT's lack of inductive biases—the very property that gives it flexibility also means it must learn spatial relationships from scratch. While ViT shows excellent potential in learning high-quality image features, it is inferior in performance vs. accuracy gains, with the little gain in accuracy not justifying the poor run time of ViT when trained from scratch on mid-sized datasets. However, transfer learning and pre-training on large datasets largely mitigates this limitation.

#### 12.6.4 Architectural variants and improvements of ViTs

Recent years have seen efficient architectures like Swin Transformer, which introduced a hierarchical structure and shifted windows to reduce computational cost while maintaining strong performance on dense prediction tasks (266). CSWin Transformer achieved 85.4% Top-1 accuracy on ImageNet-1K, 53.9 box AP and 46.4 mask AP on COCO detection, and 52.2 mIOU on ADE20K semantic segmentation. Hybrid approaches are also emerging. ViT-CoMer injects spatial pyramid multi-receptive field convolutional features into the ViT architecture, which effectively alleviates problems of limited local information interaction and single-feature representation in ViT.

#### 12.6.5 ViT vs. CNN: fundamental differences

##### 12.6.5.1 Information processing paradigms

While CNNs start with very low-level local information and gradually build up more global structures in deeper layers, ViTs already have global information at the earliest layer thanks to global self-attention (267). This difference is profound: A CNN's approach is like starting at a single pixel and zooming out, while a transformer slowly brings the whole fuzzy image into focus. ViTs utilize a self-attention mechanism that enables the model to have a whole field of view even at the lowest layer, obtaining global representations from the beginning, while CNNs need to propagate layers to obtain global representations.

##### 12.6.5.2 Inductive biases and generalization

CNNs have an inductive spatial bias baked into them with convolutional kernels, whereas vision transformers are based on a much more general architecture, with first vision transformers beating CNNs by overcoming the spatial bias given enough data. The main advantage of ViTs is their ability to effectively capture global contextual information through the self-attention mechanism, enabling them to model long-range dependencies and contextual relationships, which can improve robustness in tasks requiring understanding global context.

##### 12.6.5.3 Computational complexity

The self-attention mechanism's quadratic complexity with respect to input size creates computational challenges. ViTs are computationally intensive, especially due to the self-attention mechanism, which has quadratic complexity with respect to the number of patches (268). However, self-attention-based models are highly parallelizable and require substantially fewer parameters, making them much more computationally efficient, less prone to overfitting, and easier to fine-tune for domain-specific tasks.

##### 12.6.5.4 Practical performance comparisons

ViTs consistently outperform CNNs when trained on large datasets due to their ability to model long-range dependencies via self-attention mechanisms (269). In medical imaging specifically, transformer-based models, particularly ViTs, exhibit significant potential in diverse medical imaging tasks, showcasing superior performance when contrasted with conventional CNN models across 36 reviewed studies (217).

### 12.7 Segment anything model (SAM): universal segmentation

#### 12.7.1 Segment anything model (SAM): universal segmentation

SAM represents a paradigm shift toward foundation models for computer vision (270). SAM's architecture comprises three components that work together to return a valid segmentation mask: an image encoder to generate one-time image embedding, a prompt encoder that embeds the prompts, and a mask decoder. SAM is a foundation model for segmentation trained on 11 million images and over 1 billion masks, composed of three primary modules: an image encoder, a prompt encoder, and a mask decoder (271). The lightweight mask decoder enables rapid inference once image embeddings are computed.

#### 12.7.2 Training data and zero-shot performance

Trained on the expansive SA-1B dataset, SAM excels in zero-shot performance, adapting to new image distributions and tasks without prior knowledge (272). SAM has been trained on a dataset of 11 million images and 1.1 billion masks and has strong zero-shot performance on a variety of segmentation tasks (271). Utilizing the extensive SA-1B dataset, comprising over 11 million meticulously curated images with more than 1 billion masks, SAM has demonstrated remarkable zero-shot performance, often surpassing previous fully supervised results (272).

#### 12.7.3 Evolution: SAM 2 and SAM 3

The SAM family has rapidly evolved. SAM 2 processes approximately 44 frames per second, making it suitable for applications requiring immediate feedback like video editing and augmented reality. SAM 2 extends the original model to video by treating images as single-frame videos and incorporating streaming memory for temporal consistency (273). The most recent iteration introduces revolutionary capabilities. SAM 3 delivers strong rare and unseen object generalization and high prompt reliability, in addition to stronger performance with thin, small, low contrast, and occluded objects compared to SAM 2 and SAM 1. SAM 3 can detect, segment, and track objects using text or visual prompts, introducing the ability to exhaustively segment all instances of an open-vocabulary concept specified by a short text phrase or exemplars. Unlike prior work, SAM 3 can handle a vastly larger set of open-vocabulary prompts, achieving 75%–80% of human performance on the SA-CO benchmark which contains 270K unique concepts, over 50 times more than existing benchmarks.

#### 12.7.4 Applications and limitations in SAM

SAM's promotable segmentation enables diverse applications. With the adaptation of SAM to medical imaging, MedSAM emerges as the first foundation model for universal medical image segmentation, consistently doing better than the best segmentation foundation models when tested on a wide range of validation tasks (274). However, SAM's inference speed is quite fast, generating a segmentation result in 50 milliseconds for any prompt in the web browser with CPU, though this assumes the image embedding is already precomputed. The quality may be insufficient for high-precision applications requiring near-pixel-perfect predictions.

### 12.8 Swin-U-Net: transformers for medical image segmentation

#### 12.8.1 Architecture design in Swin-U-Net

Swin-U-Net adapts the Swin Transformer's hierarchical architecture to medical segmentation (275). It uses hierarchical Swin Transformer with shifted windows as the encoder to extract context features, and a symmetric Swin Transformer-based decoder with patch expanding layer is designed to perform the up-sampling operation to restore the spatial resolution of the feature maps (276). The model uses 224 × 224 input images with 4 × 4 patches, creating tokenized inputs at H/4×W/4 resolution (277). The encoder applies patch merging layers for 2× downsampling while doubling feature dimensions, repeated three times (278). The decoder mirrors this structure with patch expanding layers for upsampling (279).

#### 12.8.2. Performance in medical imaging in Swin-U-Net

Swin-U-Net achieves the best performance with segmentation accuracy, with pure Transformer approaches without convolution better learning both global and long-range semantic information interactions, resulting in better segmentation results (275). Swin-Unet achieves excellent performance with an accuracy of 90.00% on the ACDC dataset, showing good generalization ability and robustness (280). Experiments on multi-organ and cardiac segmentation tasks demonstrate that the pure Transformer-based U-shaped Encoder-Decoder network outperforms those methods with full-convolution or the combination of transformer and convolution (125).

#### 12.8.3 Hybrid approaches and extensions in Swin-U-Net

The debate between pure transformers and hybrid architecture continues. Swin Pure U-Net3D vs. Swin Unet3D performance differences indicate that the convolutional module can compensate for ViT's inability to fit the image detail information well. Advanced variants show further improvements. FE-SwinUper achieves excellent performance with a Dice of 90.15% on the ACDC dataset, showing good generalization ability and robustness (280). A multi-transformer U-Net surpasses standalone Swin Transformer's Swin Unet and converges more rapidly, yielding accuracy improvements of 0.7% (resulting in 88.18%) and 2.7% (resulting in 98.01%) on COVID-19 CT scan and Chest x-ray datasets respectively (281).

#### 12.8.4 3D extensions

Swin UNETR employs MONAI and has achieved state-of-the-art benchmarks for various medical image segmentation tasks, demonstrating effectiveness even with a small amount of labeled data (282). The model was pretrained on 5,050 publicly available CT images and achieved top rankings on the BTCV Segmentation Challenge and Medical Segmentation Decathlon dataset. Swin UNETR has shown better segmentation performance using significantly fewer training GPU hours compared to DiNTS—a powerful AutoML methodology for medical image segmentation, making it practical for resource-constrained medical imaging applications (283).

### 12.9 Critical insights and future directions

#### 12.9.1 Architectural evolution

The transformer revolution in computer vision demonstrates several key principles. First, removing inductive biases increases model capacity and generalization at the cost of data efficiency (284). Second, hierarchical architectures like Swin Transformer successfully balance global and local feature extraction (285). Third, hybrid approaches combining transformers with convolutions often achieve optimal performance-efficiency trade-offs (268).

#### 12.9.2 The foundation model paradigm

SAM exemplifies the shift toward foundation models trained on massive datasets for zero-shot generalization (286). This approach democratizes computer vision by enabling practitioners to apply powerful models without task-specific training (287). However, questions remain about performance on specialized domains and the computational costs of inference.

#### 12.9.3 Medical imaging considerations

In medical imaging, pure transformer architectures like Swin-U-Net demonstrate that global context modeling significantly benefits segmentation tasks (288). The ability to capture long-range dependencies helps identify anatomical structures and pathologies that span large image regions. Yet hybrid architecture often performs better when fine detail preservation is critical.

#### 12.9.4. Computational reality

Despite theoretical advantages, practical deployment requires balancing accuracy against computational constraints. The quadratic complexity of self-attention remains a bottleneck for high-resolution images, driving research into efficient attention mechanisms and hybrid architectures that selectively apply global attention (289). The transformer-based revolution in computer vision continues to accelerate, with each generation of models—from ViT to Swin Transformers to SAM 3—pushing boundaries in performance, efficiency, and generalization. The field is converging toward flexible architectures that adaptively combine local and global processing, learned through self-supervised pretraining on vast datasets, enabling unprecedented capabilities across diverse vision tasks.

### 12.10 Specialized applications

In 3D medical imaging, recent advances in AI, especially vision-language foundation models, show promise in automating radiology report generation from complex 3D medical imaging data, with studies analyzing model architecture, capabilities, training datasets, and evaluation metrics (290). As domain-specific innovations, there are some technical analyses, such as brain tumor classification with explainable AI, stroke outcome prediction using lesion-centered approaches, lung cancer screening and detection, cardiac imaging analysis, and pathology image analysis (291).

### 12.11 Practical deployment challenges

As for current limitations, practical implementation of large models in medical imaging faces notable challenges, including scarcity of high-quality medical data, need for optimized perception of imaging phenotypes, safety considerations, and seamless integration with existing clinical workflows and equipment (292). There are some emerging solutions, including federated learning for privacy-preserving collaborative training, edge deployment for real-time analysis, integration with PACS and clinical workflows, and continuous model monitoring and updating (293).

### 12.12 Future directions

AI algorithms will not only highlight abnormalities but also suggest potential diagnoses and probabilities, with natural language processing streamlining reporting processes by automatically generating structured reports, and advanced machine learning techniques comparing patient scans against extensive databases (29). There are some emerging technologies, such as photon-counting CT, whole-body MRI, AI-enabled point-of-care devices, generative AI for patient communication, and agentic AI systems for complex workflows. As pivotal takeaways, transformers are surpassing CNNs for many medical imaging tasks, particularly when combined with proper pre-training (217). Foundation models are democratizing AI in healthcare by requiring fewer local data for fine-tuning (294). Diffusion models are emerging as superior alternatives to GANs for synthetic data generation (295). XAI is critical but must be designed with human-centered principles and validated with end users. Bias and fairness require continuous monitoring across deployment contexts, not just initial training data. Multi-modal integration (imaging + clinical + genomic) is becoming standard (296). Physics-informed approaches reduce data requirements while improving interpretability. The field is rapidly evolving toward more generalizable, interpretable, and fair AI systems that can be deployed safely in diverse clinical settings.

## 13 Conclusions

Computer vision engineers develop algorithms, software, and hardware systems that enable machines to process, analyze, and make decisions based on visual data, such as images and videos. CNNs excel at tasks such as image segmentation, feature extraction, and classification and analyze various medical images well, including x-rays, MRIs, CT scans, and ultrasound images. As technology continues evolving, computer vision in healthcare will further transform patient care, medical research, and healthcare delivery, ultimately improving health outcomes and healthcare system efficiency.

CNNs are powerful DL models that have revolutionized several fields, particularly image and video processing. CNNs automatically learn features from data, making them highly effective for tasks such as image recognition, classification, and segmentation. Nonetheless, CNNs are still evolving, and ongoing research focuses on improving their efficiency, accuracy, and applicability to diverse problem domains. As the DL field advances, CNN models may play an increasingly important role in solving complex real-world problems in various industries. The CNN architecture U-Net has revolutionized biomedical image segmentation. This powerful model is the foundation of numerous advances in medical image analysis because of its ability to segment rapidly and accurately. U-Net is a cornerstone in the development of advanced medical image segmentation techniques, driving advances in computer-aided diagnosis and precision medicine.

Foundation models are set to redefine how radiologists approach diagnostics and patient care, with these advanced systems capable of handling a wide range of tasks. Vision-language models integrate computer vision and natural language processing to address complex tasks such as disease classification, segmentation, cross-modal retrieval, and automated report generation. Transformer-based multimodal predictions consistently find that transformer models outperform typical recurrent or unimodal models.
